# Global burden of pertussis in 204 countries and territories, from 1990 to 2019: results from the Global Burden of Disease Study 2019

**DOI:** 10.1186/s12889-024-18968-y

**Published:** 2024-05-30

**Authors:** Yanwu Nie, Yu Zhang, Zhen Yang, Naibo Wang, Shengnan Wang, Yong Liu, Han Jiang, Lei Wu

**Affiliations:** 1 School of Public Health, Jiangxi Provincial Key Laboratory of Disease Prevention and Public Health, Jiangxi Medical College, Nanchang University, Nanchang, 330006 China; 2 The Fourth Affiliated Hospital of Xinjiang Medical University, Xinjiang, 830054 China; 3 Department of Cardiothoracic Surgery, The Second Affiliated Hospital of Nanchang University, Nanchang, 330006 China

**Keywords:** GBD 2019, Burden, Pertussis, Global trend, Epidemiology

## Abstract

**Objectives:**

This study aimed to examine the impact of pertussis on the global, regional, and national levels between 1990 and 2019.

**Methods:**

Data on pertussis on a global scale from 1990 to 2019 were collected from the 2019 Global Burden of Disease Study. We performed a secondary analysis to report the global epidemiology and disease burden of pertussis.

**Results:**

During the period spanning from 1990 to 2019, pertussis exhibited a steady global decline in the age-standardized incidence rate (ASIR), age-standardized disability-adjusted life years rate (ASYR), and age-standardized death rate (ASDR). Nevertheless, upon delving into an in-depth analysis of various regions, it was apparent that ASIR in southern sub-Saharan Africa, ASYR and ASDR in high-income North America, and ASDR in Western Europe and Australasia, were witnessing an upward trajectory. Moreover, a negative correlation was observed between the Socio‑demographic Index (SDI) and burden inflicted by pertussis. Notably, the incidence of pertussis was comparatively lower in men than in women, with 0–4-year-olds emerging as the most profoundly affected demographic.

**Conclusion:**

The global pertussis burden decreased from 1990 to 2019. However, certain regions and countries faced an increasing disease burden. Therefore, urgent measures are required to alleviate the pertussis burden in these areas.

**Supplementary Information:**

The online version contains supplementary material available at 10.1186/s12889-024-18968-y.

## Introduction

Pertussis, an acute respiratory infection induced by *Bordetella pertussis*, causes an array of primary symptoms, such as spasmodic cough, dyspnea, and Stridor. Its effects can persist for extended periods, stretching over weeks or months [1, 2]. Before the pertussis vaccine was widely available, the disease was a leading cause of death among children worldwide, particularly affecting infants and young children[3]. Its impact on children’s lives and well-being poses a serious threat. Vaccination is the primary intervention for controlling pertussis or for reducing its burden. Following the global implementation of the Expanded Programme on Immunization in 1974, the incidence and fatality rates of this disease have considerably diminished [4]. However, the current global disease burden of pertussis remains high and poses a serious health hazard to people, especially infants and young children. Recently, there has been a “resurgence” of pertussis despite the high uptake of pertussis-containing vaccines in children[5, 6]. Between 2008 and 2015, pertussis outbreaks were reported in numerous regions, including Brazil, Mexico, and California [7–9]. This indicated the presence of some regions where the burden of pertussis increased, thereby urging exploration of the global impact of this ailment. Surprisingly, despite this urgency, no comprehensive analysis of the global data has been conducted.

Using global data on the burden of disease caused by pertussis from 1990 to 2019 from the Global Burden of Disease Study (GBD) 2019, this study aims to scrutinize the worldwide distribution of pertussis burden, uncover the heightened incidence of the disease in both geographical regions and age cohorts, and investigate the correlation between sociodemographic index (SDI) and the burden of pertussis.

## Materials and methods

### Data resources and collection

Data for this study were obtained from the Global Health Data Exchange (http://ghdx.healthdata.org/gbd-results-tool). The origins of the data and the advanced statistical methods implemented in the GBD 2019 are explained in detail in a publication [10] that comprehensively encompasses 369 maladies and injuries as well as 87 risk factors across 204 nations [10–12]. For this study, global pertussis data from 1990 to 2019 were obtained from the GBD 2019. The data variables included sex, age, region, country, sociodemographic indices, disease incidence, mortality rates, and disability-adjusted life years (DALYs). Pertussis was defined according to the 10th revision of the International Classification of Diseases.

### Estimation of annual percentage change

The EAPC is Estimation of annual percentage change that is calculated from the natural logarithm of the fitted rate derived from the regression model and is used to assess trends in age-specific rates over a designated timeframe[13]. Specifically, the logarithmic value of the annual change in the standard age-specific rate conforms to a linear regression model as it pertains to time [14]. In this study, the EAPC was used to depict the trends in the age-standardized incidence rate (ASIR), age-standardized DALYs rate ASYR, and age-standardized death rate (ASDR) for pertussis between 1990 and 2019 as follows:

1 $$\mathrm{Ln}(\mathrm y)=\mathrm\alpha+\mathrm\beta(\mathrm x)+\mathrm\epsilon,$$

2 $$\mathrm{EAPC}=100\%\times(\exp(\mathrm\beta))-1$$

where “y” represents the rate (morbidity, mortality, etc.), “x” denotes the year, “β” stands for the slope of the trend segment, “α” symbolizes the intercept, and “ε” denotes the error term. If both the EAPC and its 95% confidence interval (CI) exceeded zero, the corresponding age-standardized rate exhibited an upward trend. Conversely, if both the EAPC and its 95% CI fell below zero, the corresponding age-standardized rate displayed a downward trend.

### SDI

The SDI serves as a gauge of the developmental status of a country or region [15]. The SDI is computed by taking the geometric mean of three indicators: per capita income, average years of schooling among individuals aged ≥ 15 years, and the total fertility rate [16]. In this study, adhering to the classification standards delineated by the GBD 2019, the SDI was partitioned into five distinct categories: low (< 0.46), moderately low (0.46–0.60), moderately high (0.61–0.69), significantly high (0.70–0.81), and elevated (> 0.81). Notably, a lower SDI indicated a reduced level of social development [17].

### Statistical analysis

In this study, ASIR, ASYR, ASDR and the 95% uncertain intervals (95% UI) were used to demonstrate the global burden and epidemic characteristics of pertussis, grouped by SDI, gender, age group, country and region. EAPC was used to estimate the change trend and rate of ASIR, ASYR and ASDR in each region during 1990–2019. If normal distribution was followed, Pearson correlation was used, otherwise Spearman rank correlation was used to investigate the correlation between SDI and ASIR, ASYR, ASDR, EAPC of ASIR, EAPC of ASYR, EAPC of ASDR. R (version 4.3.1) was used for statistical analysis, and GraphPad (version 9.0) was used for graphical visualization.

## Results

### Trends in the global burden of pertussis

Between 1990 and 2019, the global annual incidence number of pertussis declined from 33,072,793.7 (95% UI: 24,973,269–42,320,661.1) to 19,519,182 (95% UI: 14,932,850.3–24,811,999.2), representing a reduction of 40.98%. Concomitantly, the ASIR for pertussis decreased from 618.2 (95% UI: 466.8–791.1, per 100,000) in 1990 to 252.3 (95% UI: 193–320.7, per 100,000) in 2019, marking a decline characterized by an EAPC of -1.83% (95% UI: -1.92–1.74). Moreover, the DALYs associated with pertussis displayed a diminishing pattern of 54.53%, concurrent with an EAPC of -2.56% (95% UI: -2.68–2.44%). The mortality rates attributed to pertussis, along with the corresponding ASDRs, similarly exhibited a downward trajectory, showing a decrease of 50.60% with an EAPC of -2.57% (95% UI: -2.7–-2.45%) (Table S1–2 and Table 1).

**Table 1:** The EAPC of ASIR, ASYR and ASDR for pertussis by region from 1990 to 2019

Location	ASIR	ASYR	ASDR
Global	-1.83 (-1.92–1.74) ^a^	-2.56 (-2.68–2.44) ^a^	-2.57 (-2.7–2.45) ^a^
High SDI	-2.28 (-2.7–1.85) ^a^	-6.15 (-6.42–5.88) ^a^	-7.01 (-7.3–6.72) ^a^
High-middle SDI	-3.25 (-3.59–2.92) ^a^	-6.11 (-6.25–5.96) ^a^	-6.2 (-6.36–6.03) ^a^
Middle SDI	-1.89 (-2.02–1.75) ^a^	-4.12 (-4.25–3.98) ^a^	-4.16 (-4.29–4.02) ^a^
Low-middle SDI	-2.34 (-2.51–2.17) ^a^	-3.57 (-3.76–3.39) ^a^	-3.59 (-3.78–3.41) ^a^
Low SDI	-1.81 (-1.94–1.69) ^a^	-2.59 (-2.68–2.49) ^a^	-2.6 (-2.69–2.5) ^a^
Andean Latin America	-1.73 (-2.03–1.43) ^a^	-5.06 (-5.37–4.76) ^a^	-5.11 (-5.42–4.8) ^a^
Australasia	-1.97 (-2.57–1.37) ^a^	-0.91 (-1.47–0.34) ^a^	9.65 (8.28–11.03) ^a^
Caribbean	-1.12 (-1.24–0.99) ^a^	-1.6 (-1.69–1.51) ^a^	-1.61 (-1.71–1.52)^a^
Central Asia	-3.43 (-3.91–2.94) ^a^	-4.46 (-4.9–4.02) ^a^	-4.51 (-4.96–4.06) ^a^
Central Europe	0.15 (-0.52–0.83)	-4.2 (-4.89–3.51) ^a^	-4.64 (-5.31–3.95) ^a^
Central Latin America	-1.24 (-1.86–0.61) ^a^	-4.68 (-5.57–3.79)^a^	-5.21 (-6.12–4.28) ^a^
Central Sub-Saharan Africa	-1.8 (-2.1–1.5) ^a^	-2.88 (-3.13–2.63) ^a^	-2.89 (-3.14–2.64) ^a^
East Asia	-6.55 (-7.33–5.77) ^a^	-11.05 (-11.72–10.37) ^a^	-11.15 (-11.83–10.48) ^a^
Eastern Europe	-5.37 (-7.32–3.37) ^a^	-4.97 (-6.51–3.4)^a^	-3.65 (-4.36–2.94) ^a^
Eastern Sub-Saharan Africa	-1.75 (-1.91–1.59) ^a^	-2.75 (-2.89–2.62) ^a^	-2.76 (-2.9–2.62) ^a^
High-income Asia Pacific	-5.72 (-6.62–4.8) ^a^	-9 (-9.7–8.3) ^a^	-9.41 (-10.11–8.71) ^a^
High-income North America	0.37 (-0.24–0.98)	0.97 (0.51–1.42) ^a^	2.91 (1.98–3.84) ^a^
North Africa and Middle East	-1.85 (-2.02–1.68) ^a^	-3.02 (-3.18–2.86) ^a^	-3.04 (-3.2–2.88) ^a^
Oceania	-1.02 (-1.25–0.78) ^a^	-1.1 (-1.33–0.88)^a^	-1.11 (-1.33–0.88) ^a^
South Asia	-2.33 (-2.65–2) ^a^	-4.07 (-4.35–3.78) ^a^	-4.09 (-4.38–3.81) ^a^
Southeast Asia	-1.16 (-1.38–0.95) ^a^	-2.71 (-2.89–2.52) ^a^	-2.73 (-2.91–2.55) ^a^
Southern Latin America	-0.97 (-1.4–0.54) ^a^	-0.1 (-0.55–0.36)	0.19 (-0.52–0.89)
Southern Sub-Saharan Africa	0.81 (0.6–1.03) ^a^	0.1 (-0.14–0.33)	0.09 (-0.15–0.32)
Tropical Latin America	-6.29 (-9.17–3.31) ^a^	-3.25 (-4.04–2.45) ^a^	-2.93 (-3.56–2.28) ^a^
Western Europe	-3.74 (-4.2–3.28) ^a^	-2.44 (-2.82–2.06) ^a^	1.16 (0.18–2.15) ^a^
Western Sub-Saharan Africa	-1.33 (-1.48–1.19) ^a^	-1.88 (-2.09–1.68) ^a^	-1.89 (-2.1–1.69) ^a^

^a^Indicates that the EAPC was statistically significant

### Burden of disease at different SDI levels

**Fig. 1:**
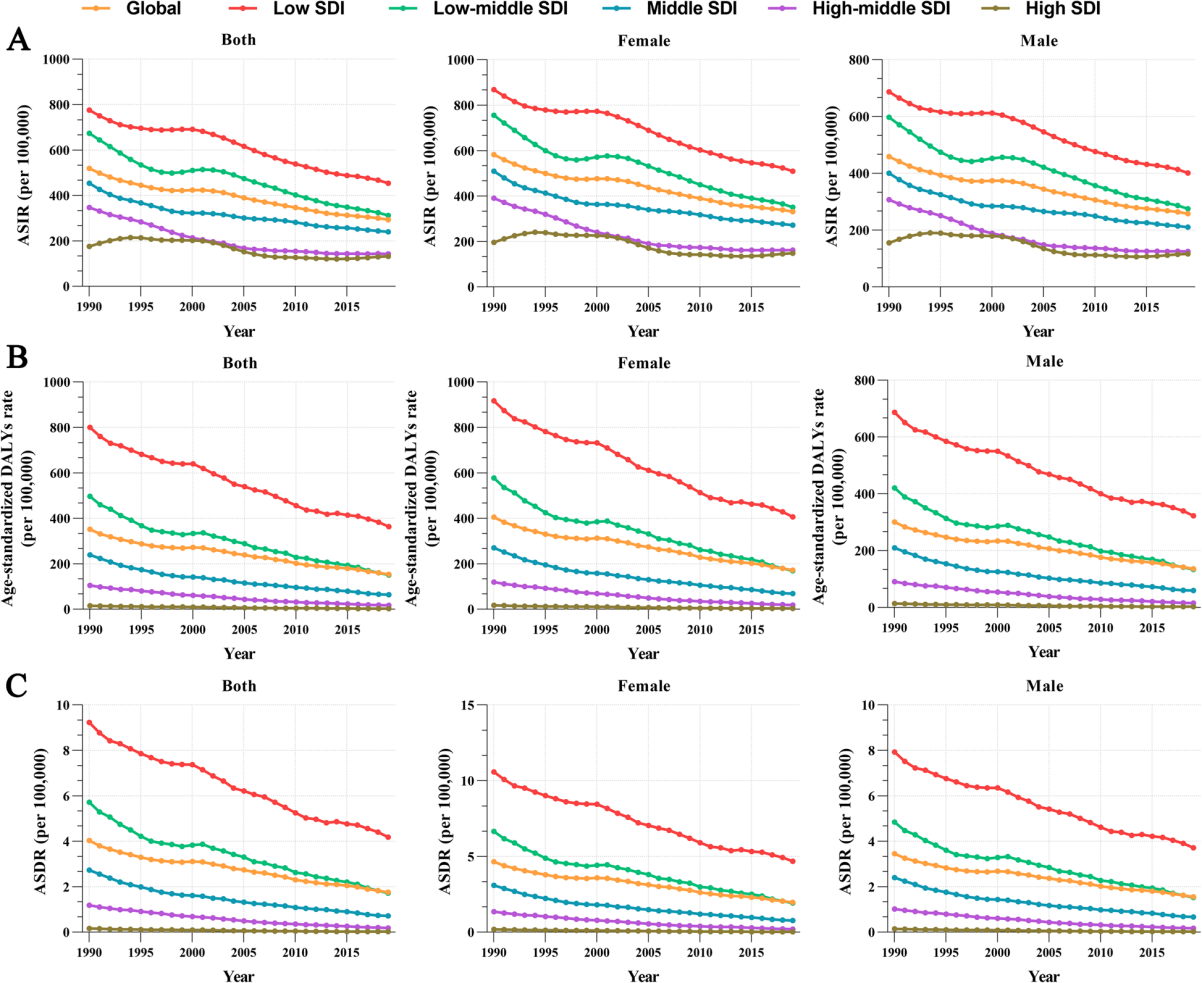
ASIR (**A**), ASYR (**B**), and ASDR (**C**) of whooping cough in different sex and SDI regions from 1990 to 2019. ASIR, age-standardized incidence rate; ASYR, Age-standardized DALY rate; ASDR, age-standardized death rate; DALYs, disability-adjusted life years; SDI, sociodemographic index

As shown in Fig. 1, the ASIR, ASYR, and ASDR pertinent to pertussis demonstrated a conspicuous downward trend, both on a global scale and within diverse SDI classifications from 1990 to 2019. Additionally, this downward trajectory remained predominantly consistent for both sexes, although a higher disease burden was observed in women than in men. We also found that SDI was negatively associated with the aforementioned rates, signifying that as the SDI escalated, the ASIR, ASYR, and ASDR associated with pertussis experienced a corresponding decline. Notably, the lowest recorded ASIR, ASYR, and ASDR across divergent SDI classifications in 2019 were 69.2 (95% UI: 52.9–89.7, per 100,000), 1.4 (95% UI: 0.8–2.3, per 100,000), and 0 (95% UI: 0–0, per 100,000), respectively, for regions characterized by a high SDI. Conversely, areas characterized by a low SDI demonstrated the highest ASIR, ASYR, and ASDR, with values reaching 692.9 (95% UI: 526.2–888.6, per 100,000), 555 (95% UI: 235.4–1057.7, per 100,000), and 6.4 (95% UI: 2.7–12.2, per 100,000), respectively. When comparing the ASIR and ASYR values of low SDI with those of a high SDI, it became apparent that the former exceeded the latter by factors of 10.01 and 396.43, respectively (Table S2).

### Burden of disease in different regions

In relation to diverse geographical regions, western sub-Saharan Africa emerged as the locus with the highest ASIR, ASYR, and ASDR, as well as the greatest number of morbidities, DALYs, and fatalities in 2019 (Table S1). A temporal analysis spanning 1990–2019 revealed a declining pattern for the majority of ASIRs, DALYs, and ASDRs across various regions worldwide. Notably, the remarkable speed of decrease observed in the three cardinal indicators noted in East Asia were -6.55% (95% UI: -7.33–-5.77%), -11.05% (95% UI: -11.72–-10.37%), and -11.15% (95% UI: -11.83–-10.48%), respectively. It is imperative to underscore that the ASIR in southern sub-Saharan Africa showed an upward trajectory characterized by an EAPC of 0.81% (95% UI: 0.6–1.03%). Similarly, the ASYR and ASDR in high-income North America showed an upward trend, with an EAPC of 0.97% (95% UI: 0.51–1.03%) and 2.91% (95% UI: 1.98–3.84%), respectively. The EAPC for the ASDR in Western Europe demonstrated an ascending pattern, with an EAPC of 1.16% (95% UI: 0.18–2.15%). Australasia had a notably high EAPC for an ASDR of 9.65% (95% UI: 8.28–11.03%) (Table 1).

### Burden of disease at 204 country levels

**Fig. 2:**
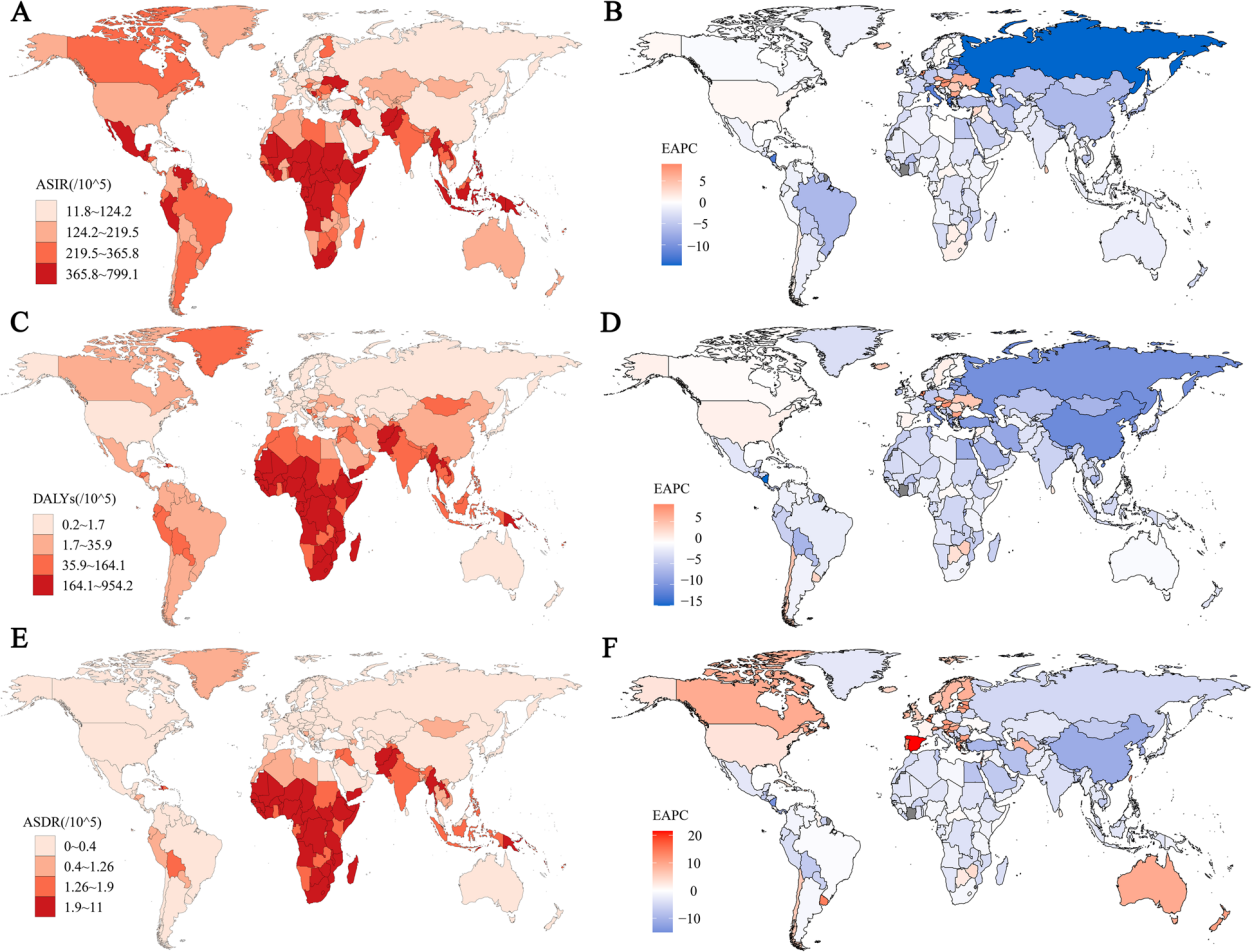
**A** ASIR of whooping cough by country in 2019. **B** EAPC of ASIR by country from 1990 to 2019. **C** DALYs of whooping cough by country in 2019. **D** EAPC of DALYs by country from 1990 to 2019. **E** ASDR of whooping cough by country in 2019. (F) ASDR of DALYs by country from 1990 to 2019. ASIR, age-standardized incidence rate; DALYs, disability-adjusted life years; ASDR, age-standardized death rate; EAPC, estimated annual percentage change

Figure 2 shows the worldwide dispersion of the ASIR, ASYR, and ASDR of pertussis along with their EAPCs, encompassing 204 nations. A discernible pattern emerges from the figure, accentuating Africa, South Asia, Southeast Asia, and South America as clusters harboring countries with an elevated pertussis ASIR. Notably, Somalia emerged as the apex, having the highest ASIR of 799.14 per 100,000 individuals (95%UI:598.34–1018.99). Subsequently, the Central African Republic, Equatorial Guinea, Chad, and Angola exhibited ASIRs exceeding 700 per 100,000 individuals. Additionally, our observations revealed an upward trajectory in ASIRs across 38 nations, with the most substantial growth materializing in Slovakia, showing an EAPC of 8.96% (95% UI: 8.12–9.82%) (Table S3). Notably, 21% of these countries are developed. Analogous to ASIR, nations harboring elevated ASYRs predominantly inhabit Africa, South Asia, and Southeast Asia, with Somalia once again standing out, with an ASYR of 953.97 per 100,000 individuals (95% UI: 114.57–2869.82). Among the 29 countries experiencing an upsurge in the ASYR, the Netherlands demonstrated fastest escalation, with an EAPC of 9.12% (95% UI: 8.24–10.01%). Within this category, developed countries accounted for 41.38% of the total (Table S3). Similarly, nations experiencing heightened ASDRs primarily lie in Africa, South Asia, and Southeast Asia, with Somalia exhibiting the highest ASDR at 10.99 per 100,000 individuals (95% UI: 1.26–33.36). Among the 54 countries that registered an upward trajectory in ASDRs, Spain showed the most pronounced surge, with an EAPC of 21.53% (95% UI: 18.79–24.34%). Developed countries accounted for 51.85% of the 54 countries where ASDR increased (Table S3).

### Correlation between SDI and pertussis disease burden

**Fig. 3:**
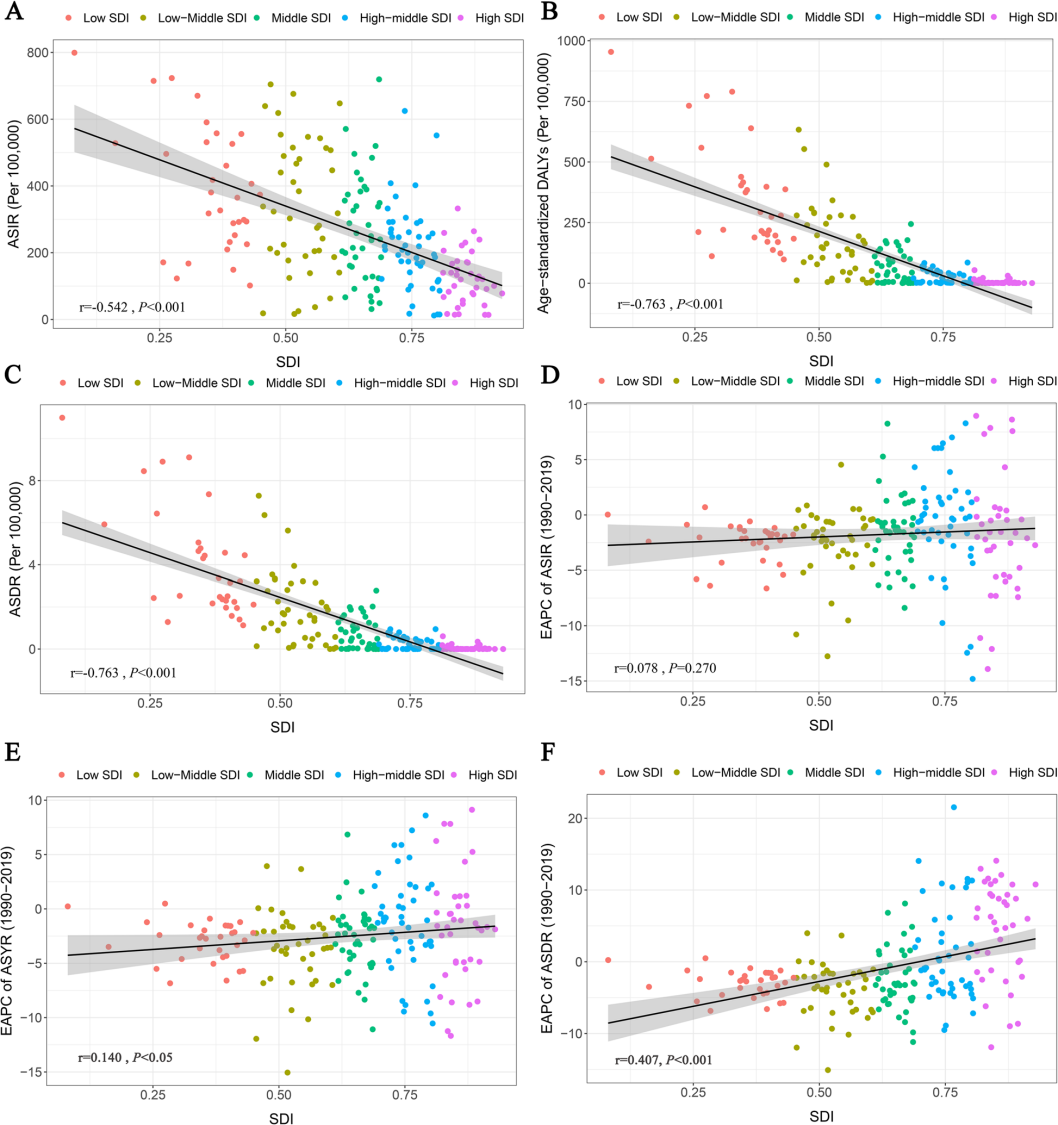
The correlation between ASIR (**A**), age-standardized DALYs rate (**B**), and ASDR (**C**) and SDI in 204 countries. The correlation between the EAPC of ASIR (**D**), the EAPC of Age-standardized DALY rate (**E**), and the EAPC of ASDR (**F**) and SDI. ASDR, age-standardized death rate; ASIR, age-standardized incidence rate; DALYs, disability-adjusted life years; EAPC, Estimated annual percentage change; SDI, sociodemographic index

Table S4 shows the normal distribution test of SDI and pertussis disease burden indicators. We found that the *P*-values were less than 0.05, indicating that the indicators did not follow normal distribution. Therefore, Spearman rank correlation was adopted in the subsequent correlation analysis. Figure 3 illustrates the relationships between the SDI and ASIR, ASYR, and ASDR. Globally, countries with an elevated SDI exhibited lower ASIR, ASYR, and ASDR than those with a lower SDI (Fig. 3). Spearman correlation analysis confirmed the negative correlation between SDI and ASIR (*r* = -0.542, *P* < 0.001, Fig. 3A), and a corresponding negative correlation between the SDI and DALYs (*r* = -0.763, *P* < 0.001, Fig. 3B). Furthermore, the data showed a comparable negative correlation between the SDI and ASDR (*r* = -0.763, *P* < 0.001, Fig. 3C). Additionally, an affirmative correlation was observed between the SDI and EAPC of ASYR and ASDR using correlation coefficients of *r* = 0.140 and 0.407, respectively (*P* < 0.05, Fig. 3E, F). However, no discernible correlation was observed between the SDI and EAPC of the ASIR (Fig. 3D).

### Age-specific distribution of disease burden for pertussis

**Fig. 4:**
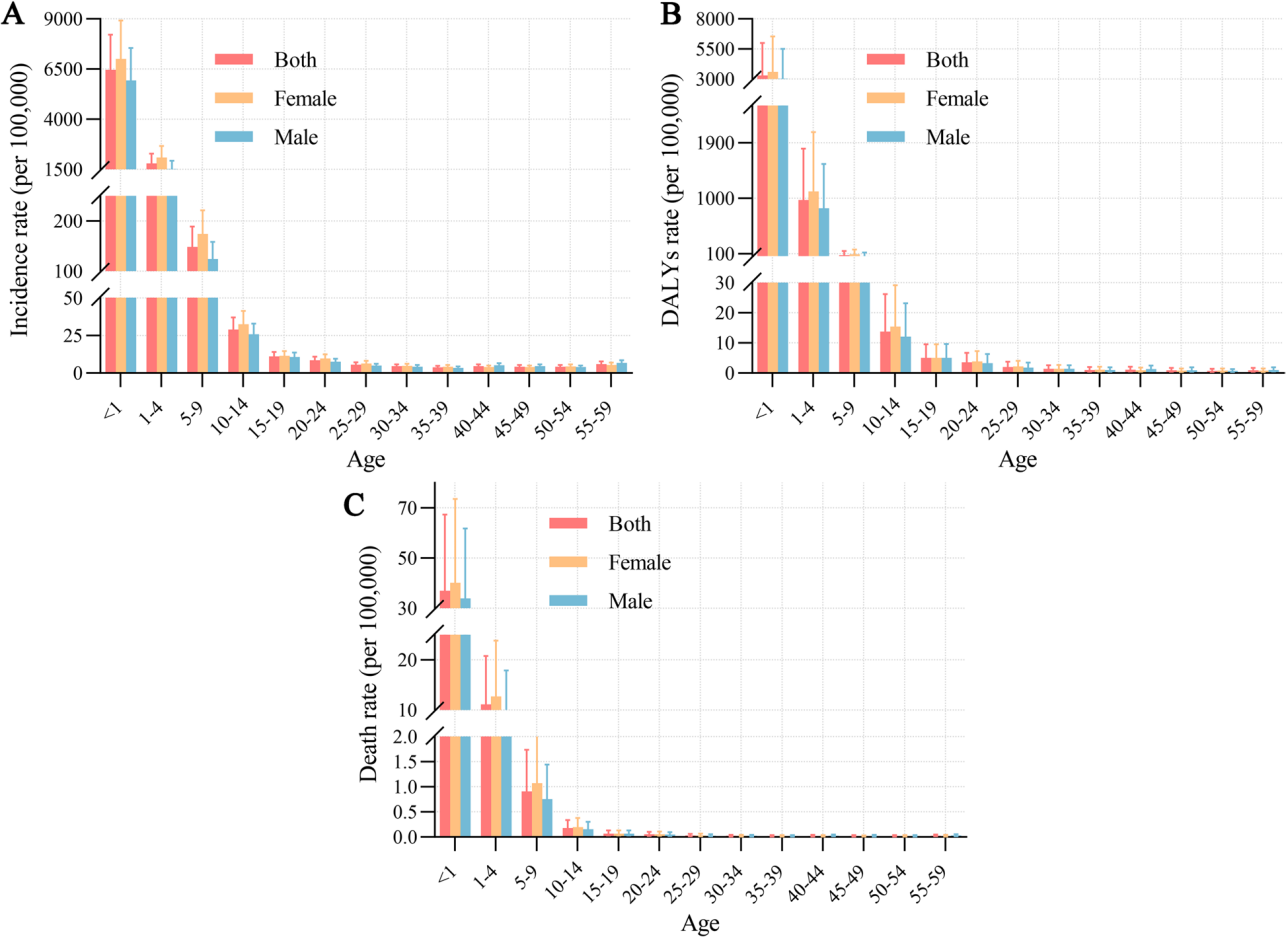
The incidence (**A**), DALYs (**B**), and death (**C**) rate of tetanus by age and sex in 2019. DALYs, disability-adjusted life years

We analyzed the incidence, DALYs, and mortality attributed to pertussis across various age cohorts. Our findings revealed that the age group under one year exhibited a pronounced incidence and mortality rate, with the highest disease burden. Subsequently, the burden of pertussis demonstrated a notable decline with age, with 1–4-year-olds marking the next tier of disease burden (Fig. 4). Amalgamating the age groups, we scrutinized the distribution of illness cases within the four age categories (< 1, 1–4, 5–9, and ≥ 10) across different SDI levels. We noted a slight difference in the composition of the age groups, with areas with low SDI having a higher proportion of cases in the < 1 year age group (Figure S1).

## Discussion

To date, we have yet to discover any researchers who have conducted a comprehensive study on the global disease burden of pertussis across 204 countries and territories. We found that the global pertussis ASIR, ASYR, and ASDR decreased over the course of the preceding three decades. Africa, South Asia, and Southeast Asia had a heavier burden of pertussis. Nevertheless, the ASIR, DALYs, and ASDR of pertussis exhibited variations across diverse countries and regions. Several indicators demonstrated upward trajectories in many nations, including developed countries. Moreover, a discernible negative correlation was observed between the SDI and the burden imposed by pertussis. Women and children aged 0–4 years emerged as cohorts with a large burden of pertussis.

Globally, the burden of pertussis exhibited a downward trajectory from 1990 to 2019, which can be attributed to the implementation of expanded program of immunization, a global strategy[18]. With the advent of the 1980s and beyond, widespread adoption and prolonged use of vaccines across most parts of the world have bolstered childhood vaccine coverage, thereby safeguarding a number of children from vaccine-preventable diseases [19]. Persistent endeavors to enhance vaccine coverage assume a pivotal role in the battle against diseases that can be prevented by immunization [20].

Both SDI stratification trend and Spearman correlation analysis showed a negative correlation between pertussis disease burden and SDI. Countries with lower SDI scores had a greater incidence of pertussis, higher DALY rates, and increased mortality than those with higher SDI scores. Similar patterns were observed for rabies and tetanus [21, 22]. Remarkably, countries with a heightened pertussis burden in this study were predominantly concentrated in Africa, South Asia, and Southeast Asia. This could be attributed to the fact that regions with elevated SDI scores boast superior public health educational systems, enhanced healthcare provisions, and offer greater opportunities for their populace to benefit from advanced healthcare systems and policy prioritization [23].

It is worth mentioning that disparate regions, specifically southern sub-Saharan Africa, high-income North America, Western Europe, and Australasia, witnessed an upsurge in indicators pertaining to the burden of pertussis. Possibly as a result of aggregating countries with diverse healthcare systems and surveillance capabilities, global trends and even SDI-strata trends do not seem to show this phenomenon. Factors contributing to the resurgence of pertussis remain unclear, with variations in the definition and diagnostic criteria for pertussis, vaccination duration, and immunization levels potentially influencing its occurrence and spread [24]. A study on child vaccination in sub-Saharan Africa showed that compared with the global average, child immunization rates in sub-Saharan Africa are low [25], and lower vaccination rates are more likely to cause outbreaks of pertussis. However, high-income countries may have higher vaccine coverage ratios and more complete vaccination programs that theoretically reduce disease risk, but they also have higher health care resources and better surveillance systems, which in turn can increase the likelihood of detecting cases and thus increase the incidence measured.

Among the 204 nations worldwide, 38, 29, and 54 countries displayed an upward trend in the ASIR, ASYR, and ASDR of pertussis, respectively. Notably, 21.05%, 41.38%, and 51.85% of these countries experiencing an increase were classified as developed countries. This suggests that pertussis prevention and control should be strengthened not only in developing countries, but also in developed regions. At the same time, it is expected that the relevant departments can carry out investigations in the areas with a high burden of pertussis or an increasing burden to identify the specific reasons.

We also found a slight discrepancy in the pertussis burden between women and men, consistent with the reports of Ballayira et al. in Mali and Alamaw et al. in Ethiopia [26]. Furthermore, we observed a distinct susceptibility to pertussis in 0–4-year-olds. This may be because parents are more concerned about the health of infants and young children, infants with low immunity are more likely to show the typical symptoms of pertussis, and are more likely to be diagnosed by doctors. In addition, pertussis vaccine requires 3 doses to produce protective antibodies [27]. Therefore, late completion of vaccination with pertussis components is also responsible for the higher disease burden in children aged 0–4 years.

To the best of our knowledge, this would be the first study to reveal the global epidemiological trends and characteristics of pertussis. Our study presented a comprehensive depiction of disease burden, meticulously stratified by age, sex, and SDI. Additionally, it provided a nuanced analysis of disease incidence across different strata, worldwide, regionally, and nationally. However, some limitations in this study must be acknowledged. Similar to most GBD publications [28, 29], our findings rely heavily on mathematical models to assess the burden of disease across countries. This will lead to errors in GBD data estimation, affecting the quality, accuracy and comparability of our results [10]. Pertussis is underdiagnosed and under-informed in most age groups in many countries, especially among older children, adolescents and adults, underestimating the burden of pertussis disease in this population. Second, due to limitations in GBD data, the age spectrum considered in our study failed to include individuals aged ≥ 59 years, thus excluding older adults from our study. Finally, due to the limitations of data acquisition, it is difficult to explore the influencing factors and find the specific reasons for the inconsistent disease burden in different regions.

## Conclusion

The global burden of pertussis generally showed a downward trend from 1990–2019. However, several countries still showed upward trends. Regions with a low SDI, particularly Africa, had a high pertussis burden, and thus urgently need measures to reduce it.

### Supplementary Information

Supplementary Material 1.

Supplementary Material 2.

Supplementary Material 3.

Supplementary Material 4.

Supplementary Material 5.

## Data Availability

All the data are available from the Global Health Data Exchange query tool ( http://ghdx.healthdata.org/gbd-results).
