# Supplementary material for: Global burden of pertussis in 204 countries and territories, from 1990 to 2019: results from the Global Burden of Disease Study 2019
Source: BMC Public Health. 2024 May 30;24:1453. doi: 10.1186/s12889-024-18968-y (PMC11141049; doi:10.1186/s12889-024-18968-y)
Supplement: Supplementary file 1 — Supplementary Material 1. [file 12889_2024_18968_MOESM1_ESM.docx]

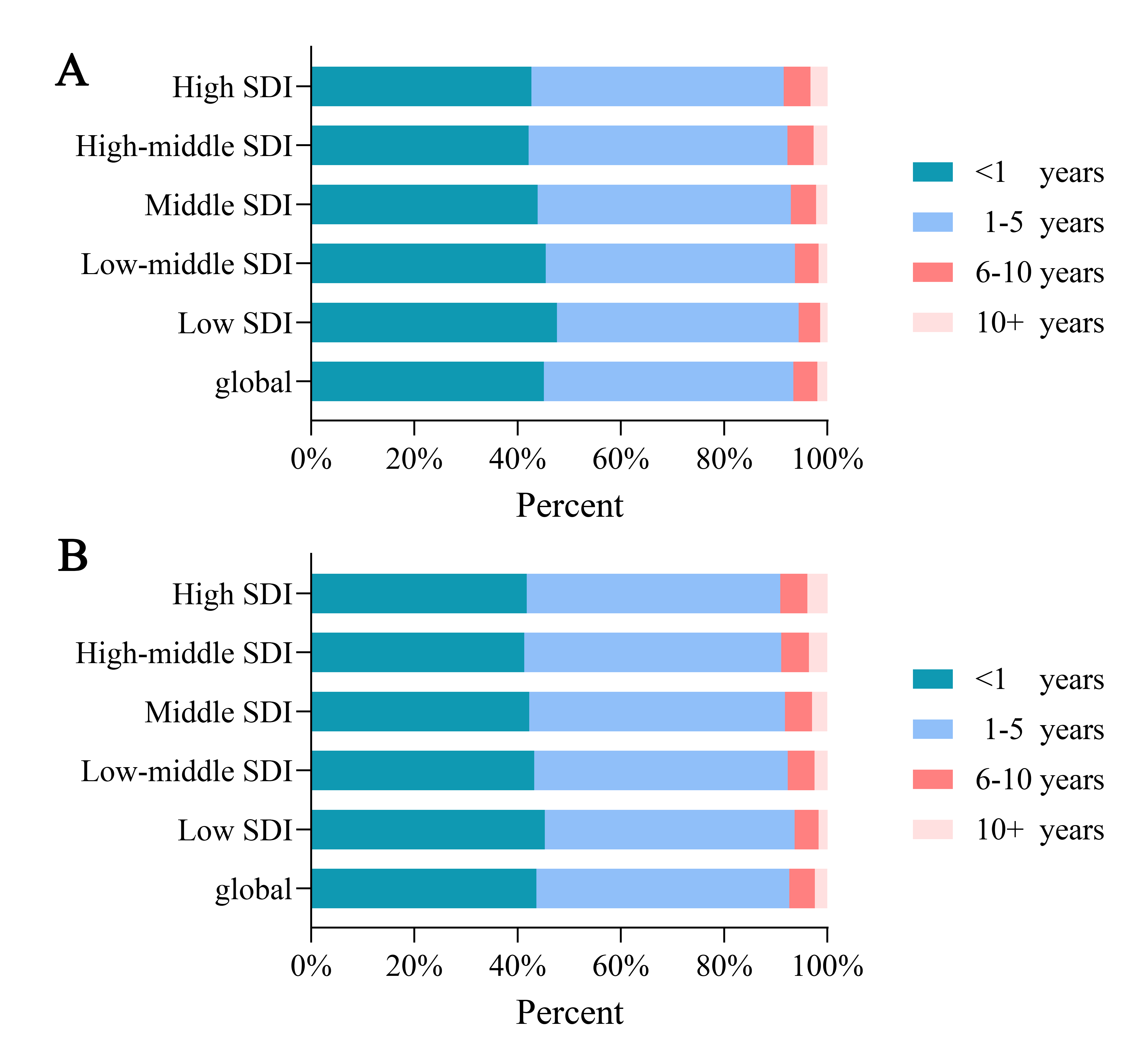


**Figure S1** Age ratio of incidence of pertussis disease by different SDI classifications in 1990 (A) and 2019 (B).
