# Supplementary material for: Global burden of pertussis in 204 countries and territories, from 1990 to 2019: results from the Global Burden of Disease Study 2019
Source: BMC Public Health. 2024 May 30;24:1453. doi: 10.1186/s12889-024-18968-y (PMC11141049; doi:10.1186/s12889-024-18968-y)
Supplement: Supplementary file 2 — Supplementary Material 2. [file 12889_2024_18968_MOESM2_ESM.docx]

Table S1. The number of incidence, DALYs, and Death of pertussis in 1990 and 2019 for all regions

| Location |  | 1990 | | |  |  | 2019 | | |  |
| --- | --- | --- | --- | --- | --- | --- | --- | --- | --- | --- |
|  | Incidence | | DALYs | Death | | Incidence | | DALYs | Death | |
| Global | 33072793.7  (24973269-42320661.1) | | 22416939  (9353255.4-44895843) | 256638.1  (105977.7-516721.8) | | 19519182  (14932850.3-24811999.2) | | 10192027  (4736515.3-18481643.3) | 116510.3  (53501.7-212098) | |
| High SDI | 1025150  (783415.8-1309850.5) | | 88133.9  (32252.9-186759.1) | 940.7  (297.8-2049.2) | | 701074  (536415.7-909458.8) | | 14298.2  (8333.4-23768.3) | 111.2  (54.9-213.7) | |
| High-middle SDI | 3618051.8  (2770424.6-4601511.3) | | 1083597.8  (367144.6-2444239.4) | 12253.9  (4037.2-28028.8) | | 1175724.8  (897061.2-1505172.2) | | 138286.7  (62590.3-280615.5) | 1518.2  (640.6-3182.4) | |
| Middle SDI | 9316131.9  (7083510.6-11956028.1) | | 4909055.9  (1951313.3-9885004.7) | 56112.5  (22105.1-113788) | | 4413957.3  (3385896.5-5623201.6) | | 1164802.9  (556437.3-2133906.4) | 13204  (6117.8-24455.8) | |
| Low-middle SDI | 11437453.3  (8611012.5-14687446.4) | | 8437579.2  (2764937.2-19890058) | 96729.3  (31253.4-229080.7) | | 5392662.7  (4125239.1-6860954.8) | | 2600932.1  (1111747.1-5244151.6) | 29735  (12588.2-60449.5) | |
| Low SDI | 7657776.4  (5750227.2-9778300.3) | | 7885198.8  (2993817.8-16010379.5) | 90448.6  (34205.7-184353.3) | | 7820421.1  (5938978.3-10028952) | | 6264491  (2656567.6-11938447.7) | 71836.4  (30237.5-137451.6) | |
| Andean Latin America | 280268.6  (212572-358840.1) | | 225776.7  (64720.2-530792.9) | 2585.6  (726.9-6095.9) | | 210040.9  (160674.3-267268) | | 62461.7  (21600.3-139220.4) | 707.2  (232.2-1600.8) | |
| Australasia | 32673.4  (24986.4-41717.2) | | 227.7  (131.6-360.4) | 0.1  (0-0.1) | | 24985.5  (19097.7-32375.4) | | 217.3  (132.8-333.2) | 0.5  (0-1.1) | |
| Caribbean | 192194.1  (145812.5-246264.8) | | 149366.1  (30205.3-452248.9) | 1710.5  (328.6-5205.6) | | 134113.2  (102515.7-170661.1) | | 80694.4  (17161.1-224915.2) | 922.7  (188.6-2585.4) | |
| Central Asia | 353525.1  (269232.1-451685.9) | | 64748.7  (19454.9-155713.3) | 722.4  (210.3-1770.6) | | 173453.3  (132824.6-223354.8) | | 24198.2  (7337.8-56245.9) | 267.7  (72.2-632.8) | |
| Central Europe | 137470.4  (105301.3-177021.8) | | 16603.7  (6628.4-31648.2) | 182  (66.1-353.4) | | 102148.4  (78093.5-131247) | | 4227.5  (2139.2-7294.2) | 41.3  (18.1-78.1) | |
| Central Latin America | 1254925.7  (946906.9-1606828.1) | | 123942.6  (70997.9-223981.7) | 1333.2  (744.2-2478.4) | | 809238  (617630.6-1033141.7) | | 27047.5  (13138.7-60152) | 250.6  (100-630.1) | |
| Central Sub-Saharan Africa | 866256.6  (650125.6-1105132.9) | | 914797.3  (198416.7-2604828) | 10471.5  (2239.1-29856.7) | | 1111804.9  (840351.8-1421419.3) | | 779083.5  (195302.6-1977606) | 8922.6  (2161.9-22776.1) | |
| East Asia | 4230552.2  (3239966.2-5384659.7) | | 2735447.8  (325621.7-7667418.8) | 31316.6  (3459.8-88167.9) | | 409408.5  (300001.6-551452.3) | | 61480.7  (9545.9-174904.4) | 684.2  (84.4-2012.5) | |
| Eastern Europe | 880844  (668335.4-1128657.8) | | 6920.8  (4332.7-10567.9) | 10.6  (2.3-12.8) | | 152932.8  (116548.4-195114.8) | | 1344.6  (871.6-1967.7) | 3.5  (1.3-4.9) | |
| Eastern Sub-Saharan Africa | 2389974.2  (1799814.2-3067735) | | 2971610.5  (1000756.3-6896683.3) | 34079.2  (11375-79378.2) | | 2422348.5  (1844347.6-3096758.6) | | 2050301.3  (758035.9-4169555.1) | 23512  (8589.3-47980.4) | |
| High-income Asia Pacific | 291660.9  (223786-371620.1) | | 36509.3  (5644.4-99822.3) | 402.5  (44.9-1144) | | 65008.8  (49006.8-85100.1) | | 2335.1  (736-5823.6) | 22.2  (3.8-61.6) | |
| High-income North America | 196433.6  (149018.5-254608.7) | | 1870.3  (1270.5-2703.6) | 6  (4.7-8.6) | | 383802.8  (293185.7-495023.4) | | 3533.5  (2366.9-5181.3) | 10.5  (4.8-13.5) | |
| North Africa and Middle East | 2498695  (1900827.2-3208498.9) | | 1437902.5  (577395.9-2777134.2) | 16401.3  (6509.6-31833.3) | | 1591448.8  (1217987.4-2024072) | | 646174.8  (241547.4-1370936.2) | 7359.1  (2705.9-15695.7) | |
| Oceania | 60258.7  (45396.5-77299.7) | | 45879.4  (9608.4-130515.3) | 525.3  (107.1-1501.2) | | 92857.7  (70414.6-118838.8) | | 66721.1  (10365.2-185619.3) | 763.9  (114.3-2139.3) | |
| South Asia | 11353847.1  (8549036.2-14559966.2) | | 8851581.2  (1887197.4-23594025.6) | 101617.6  (21045.5-272526.1) | | 4825436.1  (3701370.5-6150133.6) | | 2264471.6  (584572.7-5186198.3) | 25922.4  (6523.3-59934.9) | |
| Southeast Asia | 3411906.7  (2574725.2-4365249.4) | | 1969364.9  (637850.7-4385627.8) | 22550.4  (7110.6-50288.4) | | 1983164.8  (1516604.3-2521032) | | 754825.8  (284538-1529047.4) | 8620.5  (3207.9-17613.6) | |
| Southern Latin America | 126181.6  (96803.1-160772.1) | | 3763.8  (2688.8-5530.4) | 33.1  (21.5-52.6) | | 116930.5  (89454.9-149313.6) | | 2873.2  (1806-4648.5) | 23.7  (12.6-43.7) | |
| Southern Sub-Saharan Africa | 265028.7  (202472.5-337691.8) | | 158703.2  (40773.8-417814.6) | 1815.9  (452.1-4813.5) | | 353995.6  (269340.8-453427.7) | | 153956.9  (41273.6-355263.4) | 1758.6  (448.7-4091.6) | |
| Tropical Latin America | 919136.9  (696203.9-1175031.9) | | 31147.6  (17362.8-57261.3) | 285.4  (132.5-582.3) | | 416039.8  (318889.5-531258.6) | | 11062.4  (7165.1-17082) | 94.1  (53.9-159.5) | |
| Western Europe | 505057.6  (387438-644557) | | 4607.8  (2942.9-6885.8) | 13.3  (6.6-22.3) | | 223646.5  (169961.1-289954.2) | | 2261.1  (1487.3-3256.7) | 8.6  (1.6-12.6) | |
| Western Sub-Saharan Africa | 2825902.7  (2122979.2-3611336.7) | | 2666167.4  (894452.8-5891288) | 30575.6  (10165.5-67927.2) | | 3916376.7  (2955104.8-5016571.4) | | 3192754.8  (965612.1-7675369.2) | 36614.2  (10853.2-88607.9) | |

Abbreviations: DALYs, disability-adjusted life years；SDI, Socio-demographic index.
