# Supplementary material for: Global burden of pertussis in 204 countries and territories, from 1990 to 2019: results from the Global Burden of Disease Study 2019
Source: BMC Public Health. 2024 May 30;24:1453. doi: 10.1186/s12889-024-18968-y (PMC11141049; doi:10.1186/s12889-024-18968-y)
Supplement: Supplementary file 3 — Supplementary Material 3. [file 12889_2024_18968_MOESM3_ESM.docx]

Table S2. The ASIR, ASYR, ASDR of pertussis in 1990 and 2019 for all regions

| Location |  | 1990 | | |  |  | 2019 | | |  |
| --- | --- | --- | --- | --- | --- | --- | --- | --- | --- | --- |
|  | ASIR | | ASYR | ASDR | | ASIR | | ASYR | ASDR | |
| Global | 618.2 (466.8-791.1) | | 419 (174.8-839.2) | 4.8 (2-9.7) | | 252.3 (193-320.7) | | 131.7 (61.2-238.9) | 1.5 (0.7-2.7) | |
| High SDI | 124.7 (95.3-159.3) | | 10.7 (3.9-22.7) | 0.1 (0-0.2) | | 69.2 (52.9-89.7) | | 1.4 (0.8-2.3) | 0 (0-0) | |
| High-middle SDI | 314.5 (240.8-400) | | 94.2 (31.9-212.5) | 1.1 (0.4-2.4) | | 82.2 (62.7-105.2) | | 9.7 (4.4-19.6) | 0.1 (0-0.2) | |
| Middle SDI | 542.7 (412.6-696.4) | | 285.9 (113.7-575.8) | 3.3 (1.3-6.6) | | 184.2 (141.3-234.6) | | 48.6 (23.2-89) | 0.6 (0.3-1) | |
| Low-middle SDI | 1012.5 (762.3-1300.2) | | 746.9 (244.8-1760.7) | 8.6 (2.8-20.3) | | 305.7 (233.9-388.9) | | 147.4 (63-297.3) | 1.7 (0.7-3.4) | |
| Low SDI | 1450 (1088.8-1851.5) | | 1493 (566.9-3031.5) | 17.1 (6.5-34.9) | | 692.9 (526.2-888.6) | | 555 (235.4-1057.7) | 6.4 (2.7-12.2) | |
| Andean Latin America | 734.1 (556.8-939.9) | | 591.4 (169.5-1390.3) | 6.8 (1.9-16) | | 330.3 (252.7-420.3) | | 98.2 (34-218.9) | 1.1 (0.4-2.5) | |
| Australasia | 161.1 (123.2-205.7) | | 1.1 (0.6-1.8) | 0 (0-0) | | 86 (65.7-111.4) | | 0.7 (0.5-1.1) | 0 (0-0) | |
| Caribbean | 544.9 (413.4-698.2) | | 423.4 (85.6-1282.1) | 4.8 (0.9-14.8) | | 284.3 (217.3-361.8) | | 171.1 (36.4-476.8) | 2 (0.4-5.5) | |
| Central Asia | 510.4 (388.7-652.1) | | 93.5 (28.1-224.8) | 1 (0.3-2.6) | | 185.5 (142-238.8) | | 25.9 (7.8-60.1) | 0.3 (0.1-0.7) | |
| Central Europe | 111.8 (85.6-144) | | 13.5 (5.4-25.7) | 0.1 (0.1-0.3) | | 89.4 (68.4-114.9) | | 3.7 (1.9-6.4) | 0 (0-0.1) | |
| Central Latin America | 764.6 (576.9-979) | | 75.5 (43.3-136.5) | 0.8 (0.5-1.5) | | 323.7 (247-413.2) | | 10.8 (5.3-24.1) | 0.1 (0-0.3) | |
| Central Sub-Saharan Africa | 1560.3 (1171-1990.5) | | 1647.7 (357.4-4691.7) | 18.9 (4-53.8) | | 845.2 (638.8-1080.6) | | 592.3 (148.5-1503.4) | 6.8 (1.6-17.3) | |
| East Asia | 345.3 (264.5-439.5) | | 223.3 (26.6-625.8) | 2.6 (0.3-7.2) | | 27.8 (20.4-37.5) | | 4.2 (0.6-11.9) | 0 (0-0.1) | |
| Eastern Europe | 388.9 (295.1-498.3) | | 3.1 (1.9-4.7) | 0 (0-0) | | 72.8 (55.5-92.9) | | 0.6 (0.4-0.9) | 0 (0-0) | |
| Eastern Sub-Saharan Africa | 1256.8 (946.4-1613.2) | | 1562.6 (526.3-3626.7) | 17.9 (6-41.7) | | 588.3 (447.9-752) | | 497.9 (184.1-1012.6) | 5.7 (2.1-11.7) | |
| High-income Asia Pacific | 168.1 (129-214.2) | | 21 (3.3-57.5) | 0.2 (0-0.7) | | 34.7 (26.2-45.4) | | 1.2 (0.4-3.1) | 0 (0-0) | |
| High-income North America | 69.9 (53-90.6) | | 0.7 (0.5-1) | 0 (0-0) | | 105.3 (80.4-135.8) | | 1 (0.6-1.4) | 0 (0-0) | |
| North Africa and Middle East | 724.2 (550.9-929.9) | | 416.7 (167.3-804.9) | 4.8 (1.9-9.2) | | 261.4 (200.1-332.5) | | 106.2 (39.7-225.2) | 1.2 (0.4-2.6) | |
| Oceania | 931.4 (701.7-1194.8) | | 709.1 (148.5-2017.3) | 8.1 (1.7-23.2) | | 699.4 (530.4-895.1) | | 502.6 (78.1-1398.1) | 5.8 (0.9-16.1) | |
| South Asia | 1034.4 (778.9-1326.5) | | 806.4 (171.9-2149.6) | 9.3 (1.9-24.8) | | 267.3 (205-340.7) | | 125.4 (32.4-287.3) | 1.4 (0.4-3.3) | |
| Southeast Asia | 730.9 (551.6-935.2) | | 421.9 (136.6-939.5) | 4.8 (1.5-10.8) | | 294.3 (225.1-374.2) | | 112 (42.2-226.9) | 1.3 (0.5-2.6) | |
| Southern Latin America | 254.7 (195.4-324.5) | | 7.6 (5.4-11.2) | 0.1 (0-0.1) | | 175.2 (134-223.7) | | 4.3 (2.7-7) | 0 (0-0.1) | |
| Southern Sub-Saharan Africa | 504.9 (385.7-643.3) | | 302.3 (77.7-795.9) | 3.5 (0.9-9.2) | | 450.5 (342.8-577.1) | | 195.9 (52.5-452.1) | 2.2 (0.6-5.2) | |
| Tropical Latin America | 601.2 (455.4-768.6) | | 20.4 (11.4-37.5) | 0.2 (0.1-0.4) | | 186.1 (142.6-237.6) | | 4.9 (3.2-7.6) | 0 (0-0.1) | |
| Western Europe | 131.3 (100.7-167.6) | | 1.2 (0.8-1.8) | 0 (0-0) | | 51.3 (39-66.5) | | 0.5 (0.3-0.7) | 0 (0-0) | |
| Western Sub-Saharan Africa | 1467.4 (1102.4-1875.2) | | 1384.4 (464.5-3059.1) | 15.9 (5.3-35.3) | | 858.3 (647.6-1099.4) | | 699.7 (211.6-1682) | 8 (2.4-19.4) | |

Abbreviations: ASIR, age-standardized incidence rate; ASDR, age-standardized death rate; ASYR, age-standardized DALYs rate; DALYs, disability-adjusted life years；SDI, Socio-demographic index.
