# Supplementary material for: Global burden of pertussis in 204 countries and territories, from 1990 to 2019: results from the Global Burden of Disease Study 2019
Source: BMC Public Health. 2024 May 30;24:1453. doi: 10.1186/s12889-024-18968-y (PMC11141049; doi:10.1186/s12889-024-18968-y)
Supplement: Supplementary file 4 — Supplementary Material 4. [file 12889_2024_18968_MOESM4_ESM.docx]

Table S3.The incidence, death, and DALYs of pertussis in 2019 for all countries, with EAPC from 1990 and 2019

| Location |  | Incidence | |  |  |  | DALYs | |  | | Deaths | |  |
| --- | --- | --- | --- | --- | --- | --- | --- | --- | --- | --- | --- | --- | --- |
|  | EAPC | | ASIR | | EAPC | | | ASYR | | EAPC | | ASDR | |
| Afghanistan | -1.27  (-1.82--0.71) | | 531.13  (401.41-679.07) | | -2.66  (-3.27--2.05) | | | 438.8  (39.74-1347.1) | | -2.67  (-3.28--2.06) | | 5.06  (0.43-15.53) | |
| Albania | -3.28  (-4.49--2.07) | | 87.47  (65.64-114.77) | | -5.08  (-6.36--3.79) | | | 17.06  (2.42-50.57) | | -5.12  (-6.4--3.83) | | 0.19  (0.02-0.58) | |
| Algeria | -1.38  (-2.44--0.32) | | 213.03  (162.56-272.58) | | -3.23  (-4.33--2.13) | | | 54.76  (6.87-158.55) | | -3.27  (-4.36--2.17) | | 0.62  (0.06-1.82) | |
| American Samoa | 0.65  (0.12-1.19) | | 292.34  (224.24-372.64) | | -0.82  (-1.41--0.23) | | | 83.39  (11.8-245.89) | | -0.84  (-1.43--0.25) | | 0.95  (0.12-2.84) | |
| Andorra | -6.65  (-7.68--5.61) | | 42.03  (31.09-56.16) | | -8.52  (-9.54--7.49) | | | 3.43  (0.61-9.61) | | -8.64  (-9.66--7.61) | | 0.04  (0-0.11) | |
| Angola | -1.01  (-1.09--0.94) | | 704.36  (529.96-901.08) | | -2.06  (-2.33--1.79) | | | 553.35  (52.82-1747.53) | | -2.08  (-2.35--1.8) | | 6.36  (0.56-20.23) | |
| Antigua and Barbuda | 6.06  (5.17-6.95) | | 173.43  (132.84-224.25) | | 5.88  (5.1-6.68) | | | 1.33  (0.8-2.05) | | 3.95  (3-4.92) | | 0  (0-0) | |
| Argentina | -1.58  (-2.07--1.1) | | 259.67  (199.18-331.48) | | -1.17  (-1.72--0.62) | | | 6.33  (3.7-10.97) | | -1.06  (-1.86--0.26) | | 0.05  (0.02-0.11) | |
| Armenia | -2.02  (-3.24--0.79) | | 186.56  (142.8-241.09) | | -1.76  (-3.1--0.41) | | | 1.45  (0.88-2.23) | | -3.06  (-6.46-0.47) | | 0  (0-0) | |
| Australia | -1.57  (-2.32--0.82) | | 133.14  (101.6-172.4) | | -0.47  (-1.18-0.24) | | | 1.17  (0.7-1.8) | | 9.45  (8.08-10.84) | | 0  (0-0.01) | |
| Austria | 0.7  (-0.38-1.79) | | 221.77  (169.63-283.17) | | 1.12  (0.14-2.1) | | | 1.66  (0.99-2.62) | | 11.27  (9.62-12.96) | | 0  (0-0) | |
| Azerbaijan | -4.09  (-4.65--3.52) | | 338.21  (259.16-430.53) | | -5.37  (-5.99--4.75) | | | 99.45  (12.84-299.69) | | -5.4  (-6.03--4.78) | | 1.12  (0.12-3.44) | |
| Bahamas | 2.05  (0.58-3.54) | | 186.29  (142.49-239.49) | | 1.88  (0.54-3.24) | | | 1.38  (0.84-2.17) | | 0.58  (-0.46-1.64) | | 0  (0-0) | |
| Bahrain | -6.56  (-7.3--5.81) | | 39.43  (28.66-53.64) | | -8.84  (-9.59--8.08) | | | 8.8  (0.97-26.37) | | -8.89  (-9.65--8.13) | | 0.1  (0.01-0.3) | |
| Bangladesh | -4.6  (-5.1--4.1) | | 199.42  (152.41-256.07) | | -6.42  (-6.81--6.02) | | | 107.32  (10.82-327.05) | | -6.44  (-6.83--6.05) | | 1.23  (0.11-3.76) | |
| Barbados | 1.14  (0.56-1.73) | | 199.79  (152.53-256.02) | | 1.21  (0.65-1.77) | | | 1.44  (0.86-2.33) | | 2.75  (2.07-3.43) | | 0  (0-0) | |
| Belarus | -9.75  (-10.83--8.67) | | 17.22  (11.94-24.53) | | -8.47  (-9.61--7.31) | | | 0.17  (0.09-0.28) | | -4.43  (-6.88--1.91) | | 0  (0-0) | |
| Belgium | -7.31  (-8.78--5.81) | | 46.72  (34.46-62.61) | | -4.83  (-5.93--3.71) | | | 0.49  (0.29-0.79) | | 14.08  (12.14-16.05) | | 0  (0-0) | |
| Belize | -0.45  (-0.67--0.23) | | 318.17  (243.51-404.92) | | -3.55  (-4.09--3) | | | 3.2  (2.05-4.88) | | -6.69  (-8.13--5.23) | | 0.01  (0.01-0.02) | |
| Benin | -1.46  (-1.79--1.12) | | 380.72  (290.84-485.14) | | -1.97  (-2.37--1.58) | | | 374.69  (39.25-1217.75) | | -1.98  (-2.37--1.59) | | 4.31  (0.43-14.06) | |
| Bermuda | -0.17  (-0.51-0.18) | | 67.31  (50.11-89.14) | | -0.34  (-0.62--0.07) | | | 0.55  (0.32-0.88) | | -1.44  (-2.58--0.28) | | 0  (0-0) | |
| Bhutan | -10.79  (-11.74--9.83) | | 18.49  (12.84-26.32) | | -11.95  (-13.2--10.67) | | | 11.82  (1.26-38.51) | | -11.96  (-13.22--10.68) | | 0.14  (0.01-0.44) | |
| Bolivia (Plurinational State of) | -4.73  (-4.99--4.46) | | 206.55  (157.87-265.25) | | -6.94  (-7.22--6.66) | | | 117.09  (12.84-357.79) | | -6.96  (-7.24--6.68) | | 1.34  (0.13-4.12) | |
| Bosnia and Herzegovina | -2.2  (-3.4--0.99) | | 368.41  (281.67-468.56) | | -4.79  (-6.08--3.49) | | | 48.9  (9.08-112.03) | | -4.88  (-6.17--3.58) | | 0.54  (0.07-1.28) | |
| Botswana | 1.27  (0.18-2.38) | | 326.07  (249.91-415.1) | | 2.45  (1.33-3.58) | | | 173.92  (16.1-530.28) | | 2.44  (1.32-3.57) | | 1.99  (0.17-6.15) | |
| Brazil | -6.54  (-9.63--3.34) | | 258.57  (198.24-330.15) | | -1.76  (-2.82--0.7) | | | 5.33  (3.84-7.27) | | -0.52  (-1.42-0.39) | | 0.04  (0.03-0.06) | |
| Brunei Darussalam | -0.84  (-1.8-0.13) | | 83.03  (62.37-108.94) | | -2.45  (-3.2--1.69) | | | 14.99  (2.2-45.17) | | -2.5  (-3.25--1.74) | | 0.17  (0.02-0.52) | |
| Bulgaria | 7.02  (5.64-8.41) | | 219.07  (167.28-280.23) | | 7.23  (5.91-8.56) | | | 1.6  (0.97-2.49) | | 10.39  (9.25-11.55) | | 0  (0-0) | |
| Burkina Faso | -5.8  (-6.42--5.18) | | 171.17  (131.16-221.4) | | -5.51  (-6.08--4.95) | | | 211.16  (16.72-689.7) | | -5.52  (-6.09--4.95) | | 2.43  (0.18-7.94) | |
| Burundi | -6.38  (-7.42--5.33) | | 122.84  (93.63-159.13) | | -6.84  (-7.74--5.93) | | | 111.69  (9.48-365.65) | | -6.84  (-7.75--5.93) | | 1.29  (0.1-4.23) | |
| Cabo Verde | -7.99  (-10--5.94) | | 24.96  (17.74-34.85) | | -9.31  (-11.28--7.31) | | | 11.15  (1.16-36.11) | | -9.33  (-11.29--7.32) | | 0.13  (0.01-0.41) | |
| Cambodia | -5.21  (-5.6--4.83) | | 212.04  (161.78-271.34) | | -6.83  (-7.25--6.4) | | | 86.95  (10.19-272.42) | | -6.85  (-7.28--6.42) | | 0.99  (0.1-3.15) | |
| Cameroon | -2.06  (-2.33--1.8) | | 440.29  (335.18-563.05) | | -2.1  (-2.44--1.75) | | | 291.31  (24.43-920.18) | | -2.11  (-2.45--1.77) | | 3.35  (0.25-10.66) | |
| Canada | -0.45  (-0.69--0.22) | | 264.21  (202.8-337.05) | | 0.29  (0-0.58) | | | 2.12  (1.31-3.24) | | 9.34  (7.37-11.34) | | 0  (0-0.01) | |
| Central African Republic | 0.7  (0.41-0.99) | | 723.15  (544.41-925.88) | | 0.49  (0.18-0.79) | | | 771.92  (61.49-2474.49) | | 0.49  (0.19-0.8) | | 8.9  (0.66-28.75) | |
| Chad | -0.89  (-0.98--0.8) | | 714.73  (537.41-913.63) | | -1.22  (-1.4--1.05) | | | 731.99  (66.16-2174.28) | | -1.21  (-1.39--1.04) | | 8.45  (0.72-25.14) | |
| Chile | 1.58  (0.38-2.8) | | 184.42  (141.06-237.63) | | 4.73  (3.38-6.1) | | | 5.62  (1.83-10.65) | | 6.15  (4.52-7.81) | | 0.05  (0.01-0.11) | |
| China | -6.43  (-7.22--5.63) | | 49  (35.86-66.07) | | -11.08  (-11.73--10.42) | | | 7.45  (0.99-21.34) | | -11.19  (-11.84--10.53) | | 0.08  (0.01-0.24) | |
| Colombia | -1.46  (-1.87--1.05) | | 214.59  (163.9-274.47) | | -1.45  (-2.17--0.73) | | | 5.89  (2.31-11.1) | | -1.4  (-2.48--0.31) | | 0.05  (0.01-0.11) | |
| Comoros | -1.24  (-1.58--0.9) | | 338.55  (259.25-430.91) | | -2.24  (-2.65--1.82) | | | 279.61  (32.29-848.84) | | -2.25  (-2.66--1.83) | | 3.21  (0.35-9.83) | |
| Congo | 0.54  (0.08-1) | | 542.89  (411.16-694.04) | | -1.66  (-2.2--1.11) | | | 274.06  (35.37-835.27) | | -1.67  (-2.21--1.11) | | 3.15  (0.38-9.73) | |
| Cook Islands | -0.31  (-0.99-0.37) | | 247.65  (189.37-316.24) | | -3.65  (-4.12--3.17) | | | 30.45  (4.36-80.83) | | -3.74  (-4.21--3.27) | | 0.34  (0.03-0.92) | |
| Costa Rica | -2.72  (-3.4--2.04) | | 92.11  (69.45-120.49) | | -1.79  (-3.03--0.54) | | | 0.99  (0.61-1.53) | | 1.57  (-1.62-4.87) | | 0  (0-0.01) | |
| Côte d'Ivoire | -1.67  (-2.49--0.85) | | 194.78  (149-250.43) | | -1.05  (-1.85--0.25) | | | 1.5  (0.89-2.39) | | 11.55  (10.23-12.89) | | 0  (0-0) | |
| Croatia | -1.56  (-3.19-0.1) | | 69.54  (51.85-92.04) | | -1.15  (-2.69-0.41) | | | 0.57  (0.33-0.9) | | 3.91  (1.25-6.64) | | 0  (0-0) | |
| Cuba | -7.28  (-8.05--6.51) | | 34.61  (25.17-47.05) | | -11.68  (-12.35--10.99) | | | 2.23  (0.46-6.38) | | -11.91  (-12.57--11.25) | | 0.02  (0-0.07) | |
| Cyprus | 7.33  (6.54-8.11) | | 140.02  (107.06-181.46) | | 7.83  (7.11-8.55) | | | 1.06  (0.64-1.7) | | 11.17  (10.07-12.27) | | 0  (0-0) | |
| Czechia | -1.67  (-1.8--1.53) | | 396.6  (302.88-505.76) | | -2.19  (-2.47--1.9) | | | 319.49  (27.08-1091.85) | | -2.19  (-2.47--1.9) | | 3.68  (0.29-12.63) | |
| Democratic People's Republic of Korea | -9.52  (-10.73--8.3) | | 36.97  (26.84-50.37) | | -10.15  (-11.43--8.85) | | | 12.03  (1.45-37.7) | | -10.16  (-11.44--8.85) | | 0.14  (0.01-0.43) | |
| Democratic Republic of the Congo | -2.49  (-2.9--2.08) | | 460.66  (350.39-590.59) | | -3.8  (-4.12--3.47) | | | 293.39  (28.48-951.69) | | -3.8  (-4.12--3.47) | | 3.37  (0.3-10.98) | |
| Denmark | -1.63  (-2.41--0.84) | | 120.73  (92.01-156.38) | | -1.31  (-2.08--0.54) | | | 0.9  (0.54-1.47) | | 8.79  (7.6-10) | | 0  (0-0) | |
| Djibouti | 0.45  (0.21-0.68) | | 639.25  (482.46-818.88) | | 0.07  (-0.33-0.46) | | | 633.18  (69.8-1679.82) | | 0.06  (-0.33-0.46) | | 7.28  (0.76-19.43) | |
| Dominica | 6.04  (4.82-7.28) | | 174.18  (133.22-224.94) | | 5.87  (4.84-6.9) | | | 69.97  (8.04-206.61) | | 5.86  (4.84-6.89) | | 0.8  (0.08-2.38) | |
| Dominican Republic | -0.99  (-1.18--0.79) | | 446.98  (340.23-571.82) | | -2.43  (-2.74--2.11) | | | 163.86  (21.1-430.67) | | -2.45  (-2.77--2.14) | | 1.86  (0.2-4.97) | |
| Ecuador | -1.55  (-2.41--0.69) | | 339.46  (259.95-431.8) | | -3.99  (-4.93--3.04) | | | 97.44  (12.1-282.97) | | -4.03  (-4.97--3.08) | | 1.1  (0.11-3.25) | |
| Egypt | -5.19  (-6.13--4.25) | | 124.67  (94.9-161.19) | | -7.71  (-8.64--6.78) | | | 29.18  (3.26-89.49) | | -7.76  (-8.68--6.83) | | 0.33  (0.03-1.03) | |
| El Salvador | -3.59  (-4.63--2.54) | | 242.55  (185.59-309.73) | | -6.62  (-7.74--5.49) | | | 60.96  (6.44-176.99) | | -6.67  (-7.79--5.54) | | 0.69  (0.06-2.04) | |
| Equatorial Guinea | -0.9  (-1.16--0.64) | | 719.35  (540.77-919.53) | | -4.96  (-5.16--4.76) | | | 244.18  (25.51-763.04) | | -5.02  (-5.21--4.82) | | 2.78  (0.25-8.84) | |
| Eritrea | -6.63  (-7.3--5.95) | | 148.97  (114.1-193.3) | | -6.59  (-7.01--6.17) | | | 170.75  (14.41-557.09) | | -6.6  (-7.01--6.18) | | 1.97  (0.15-6.45) | |
| Estonia | -13.92  (-14.9--12.92) | | 14.29  (9.83-20.46) | | -11.24  (-12.02--10.46) | | | 0.18  (0.09-0.3) | | 11.6  (10.21-13.01) | | 0  (0-0) | |
| Eswatini | -1.39  (-1.77--1.01) | | 263.71  (202.16-336.72) | | -0.84  (-1.11--0.57) | | | 203.07  (20.49-633.87) | | -0.84  (-1.11--0.57) | | 2.33  (0.22-7.37) | |
| Ethiopia | -1.1  (-1.35--0.85) | | 590.68  (445.78-756.2) | | -3.63  (-3.86--3.4) | | | 403.46  (45.03-1135.25) | | -3.64  (-3.87--3.41) | | 4.65  (0.49-13.17) | |
| Fiji | 1.8  (0.74-2.87) | | 283.04  (217.19-361.02) | | 1.6  (0.55-2.65) | | | 134.22  (13.18-387.3) | | 1.59  (0.55-2.65) | | 1.54  (0.13-4.48) | |
| Finland | -0.09  (-0.75-0.57) | | 230.35  (176.22-294.13) | | 0.24  (-0.42-0.91) | | | 1.68  (0.99-2.65) | | 8.79  (7.58-10.02) | | 0  (0-0) | |
| France | -3.23  (-4.41--2.02) | | 100.47  (75.92-130.25) | | -1.68  (-2.35--1) | | | 1.12  (0.65-1.82) | | 1.25  (0.14-2.38) | | 0.01  (0-0.01) | |
| Gabon | -1.14  (-1.98--0.3) | | 397.79  (303.63-507.87) | | -2.99  (-4.09--1.87) | | | 169.56  (17.55-511.1) | | -3  (-4.11--1.88) | | 1.94  (0.17-5.94) | |
| Gambia | -1.63  (-2.23--1.03) | | 252.37  (193.4-322.29) | | -1.54  (-2.11--0.98) | | | 218.04  (18.41-671.82) | | -1.54  (-2.11--0.98) | | 2.51  (0.19-7.75) | |
| Georgia | -0.51  (-0.88--0.14) | | 332.42  (255.18-423.31) | | -0.44  (-0.77--0.12) | | | 2.46  (1.48-3.9) | | -1.19  (-2.16--0.22) | | 0  (0-0) | |
| Germany | -4.79  (-6.54--3.01) | | 91.26  (68.74-118.62) | | -4.87  (-6.19--3.53) | | | 0.76  (0.45-1.18) | | 0.18  (-4.17-4.72) | | 0  (0-0) | |
| Ghana | -3.53  (-3.75--3.32) | | 204.66  (156.28-262.35) | | -2.92  (-3.28--2.55) | | | 140.51  (14.85-488) | | -2.93  (-3.3--2.56) | | 1.61  (0.16-5.66) | |
| Greece | -12.45  (-13.21--11.69) | | 11.8  (8-17.08) | | -8.53  (-9.26--7.8) | | | 0.26  (0.11-0.5) | | 11.05  (9.73-12.39) | | 0  (0-0) | |
| Greenland | -1.05  (-1.68--0.42) | | 168.36  (128.97-218.06) | | -3.03  (-3.61--2.44) | | | 42.09  (4.73-116.14) | | -3.07  (-3.65--2.48) | | 0.47  (0.05-1.33) | |
| Grenada | -0.64  (-1.1--0.18) | | 249.79  (191.37-318.98) | | -0.5  (-0.99-0) | | | 1.89  (1.15-2.9) | | 0.9  (-0.37-2.18) | | 0  (0-0) | |
| Guam | 1.43  (0.71-2.16) | | 206.15  (157.51-264.56) | | 1.44  (0.57-2.33) | | | 53.97  (6.84-152.02) | | 1.44  (0.56-2.34) | | 0.61  (0.06-1.75) | |
| Guatemala | -2.57  (-3.62--1.5) | | 384.06  (293.26-489.93) | | -6.76  (-8.09--5.41) | | | 19.49  (7.03-126.43) | | -7.12  (-8.48--5.75) | | 0.2  (0.06-1.41) | |
| Guinea | -1.03  (-1.32--0.75) | | 670.23  (504.49-860.59) | | -1.51  (-1.92--1.1) | | | 789.81  (65.54-2506.78) | | -1.51  (-1.92--1.1) | | 9.11  (0.71-29.03) | |
| Guinea-Bissau | -2.12  (-2.69--1.55) | | 417.96  (318.71-533.61) | | -2.74  (-3.47--2.01) | | | 384.42  (36.49-1107.66) | | -2.75  (-3.48--2.01) | | 4.43  (0.39-12.83) | |
| Guyana | -2.29  (-2.76--1.82) | | 199.29  (152.12-255.27) | | -1.06  (-1.43--0.69) | | | 2.26  (1.34-3.8) | | 1.48  (-0.57-3.58) | | 0.01  (0-0.03) | |
| Haiti | -1.94  (-2.06--1.81) | | 406.45  (310.08-518.93) | | -2.89  (-3.02--2.76) | | | 387.28  (41.59-1215.07) | | -2.9  (-3.03--2.78) | | 4.45  (0.45-13.96) | |
| Honduras | -0.28  (-1.16-0.61) | | 224.15  (171.49-286.22) | | -3.38  (-4.27--2.47) | | | 77.97  (7.44-250.84) | | -3.41  (-4.31--2.51) | | 0.89  (0.07-2.89) | |
| Hungary | 8.29  (7.48-9.11) | | 92.65  (69.87-121.16) | | 8.59  (7.81-9.39) | | | 0.73  (0.43-1.17) | | 10.36  (9.27-11.46) | | 0  (0-0) | |
| Iceland | 4.32  (3.82-4.82) | | 203.9  (155.57-260.92) | | 4.34  (3.81-4.88) | | | 1.51  (0.9-2.39) | | 4.71  (3.36-6.08) | | 0  (0-0) | |
| India | -2.26  (-2.74--1.79) | | 281.41  (215.82-358.86) | | -4.16  (-4.6--3.72) | | | 129.31  (17.03-361.42) | | -4.19  (-4.63--3.75) | | 1.48  (0.17-4.16) | |
| Indonesia | -1.5  (-1.67--1.33) | | 389.48  (297.45-496.64) | | -3.27  (-3.47--3.07) | | | 155.31  (19.43-430.64) | | -3.29  (-3.49--3.09) | | 1.77  (0.2-4.99) | |
| Iran (Islamic Republic of) | -5.8  (-6.55--5.05) | | 53.19  (39.27-71.13) | | -8.35  (-9.12--7.56) | | | 8.28  (1.05-24.08) | | -8.41  (-9.19--7.63) | | 0.09  (0.01-0.28) | |
| Iraq | 1.04  (0.7-1.38) | | 484.41  (367.78-621.3) | | -1.43  (-1.93--0.92) | | | 111.04  (12.22-317.74) | | -1.48  (-1.99--0.97) | | 1.25  (0.11-3.66) | |
| Ireland | -5.42  (-5.69--5.15) | | 137.24  (104.66-177.66) | | -4.9  (-5.17--4.63) | | | 1.04  (0.61-1.62) | | 8.32  (6.89-9.76) | | 0  (0-0) | |
| Israel | 1.15  (0.63-1.66) | | 118.66  (90.33-153.41) | | 2.25  (1.66-2.83) | | | 1.06  (0.64-1.61) | | 11.32  (9.92-12.74) | | 0  (0-0.01) | |
| Italy | -11.91  (-13.41--10.38) | | 15.05  (10.38-21.48) | | -9.61  (-11.29--7.91) | | | 0.21  (0.14-0.29) | | -3.81  (-6.48--1.06) | | 0  (0-0) | |
| Jamaica | -1.78  (-2.13--1.43) | | 123.74  (94.24-160.04) | | -1.62  (-1.97--1.27) | | | 0.89  (0.52-1.44) | | 4.87  (3.89-5.87) | | 0  (0-0) | |
| Japan | -6.02  (-7.08--4.95) | | 79.51  (59.57-104.61) | | -5.55  (-6.43--4.65) | | | 0.69  (0.45-1.03) | | -2.74  (-4.71--0.73) | | 0  (0-0) | |
| Jordan | -0.1  (-1.9-1.73) | | 208.66  (159.54-266.45) | | -2.76  (-4.37--1.12) | | | 40.04  (4.95-109.67) | | -2.83  (-4.44--1.2) | | 0.45  (0.04-1.25) | |
| Kazakhstan | -5.7  (-7.24--4.14) | | 193.22  (147.82-248.94) | | -5.43  (-6.58--4.27) | | | 1.5  (0.92-2.34) | | -3.12  (-4.95--1.25) | | 0  (0-0) | |
| Kenya | -1.31  (-1.69--0.92) | | 302.8  (232.41-385.75) | | -1.64  (-1.89--1.39) | | | 144.02  (16.25-437.71) | | -1.63  (-1.89--1.38) | | 1.65  (0.17-5.08) | |
| Kiribati | -0.74  (-0.9--0.57) | | 480.76  (365.49-617.03) | | -1.68  (-1.84--1.52) | | | 341.85  (39.92-1011.94) | | -1.68  (-1.84--1.52) | | 3.94  (0.43-11.77) | |
| Kuwait | -5.6  (-7.82--3.32) | | 57.12  (42.45-75.99) | | -4.96  (-6.97--2.9) | | | 0.44  (0.25-0.71) | | 4.44  (3.19-5.7) | | 0  (0-0) | |
| Kyrgyzstan | -2.75  (-3.17--2.33) | | 186.94  (142.92-240.96) | | -3.58  (-3.99--3.17) | | | 1.48  (0.9-2.31) | | -7.82  (-8.68--6.96) | | 0  (0-0.01) | |
| Lao People's Democratic Republic | -1.84  (-2--1.68) | | 553.73  (418.44-708.05) | | -3.7  (-3.93--3.46) | | | 289.05  (35.06-818.33) | | -3.73  (-3.96--3.49) | | 3.31  (0.36-9.44) | |
| Latvia | -11.11  (-11.96--10.26) | | 16.62  (11.5-23.72) | | -8.1  (-9.14--7.05) | | | 0.21  (0.1-0.37) | | 12.96  (11.14-14.8) | | 0  (0-0) | |
| Lebanon | 2.43  (1.03-3.85) | | 408.2  (311.42-521.17) | | -0.86  (-1.96-0.26) | | | 70.56  (9.49-189.74) | | -0.93  (-2.03-0.19) | | 0.79  (0.08-2.18) | |
| Lesotho | -1.98  (-2.81--1.14) | | 124.35  (94.57-161.05) | | -0.38  (-1.24-0.48) | | | 104.93  (9.48-335.46) | | -0.38  (-1.23-0.49) | | 1.21  (0.1-3.89) | |
| Liberia | -2.58  (-3.26--1.9) | | 326.63  (250.65-415.91) | | -5.05  (-5.83--4.26) | | | 188.91  (18.84-627.96) | | -5.06  (-5.85--4.27) | | 2.17  (0.2-7.27) | |
| Libya | 0.09  (-0.64-0.82) | | 270.46  (207.68-345.08) | | -1.06  (-1.76--0.36) | | | 60.04  (7.23-169.31) | | -1.1  (-1.8--0.39) | | 0.68  (0.07-1.95) | |
| Lithuania | -12.1  (-13.01--11.19) | | 15.72  (10.85-22.44) | | -8.6  (-9.15--8.05) | | | 0.26  (0.12-0.44) | | 9.18  (7.63-10.74) | | 0  (0-0) | |
| Luxembourg | -7.41  (-8.91--5.89) | | 14.02  (9.67-20.02) | | -1.74  (-3.17--0.28) | | | 1.01  (0.31-2.1) | | -0.04  (-1.35-1.28) | | 0.01  (0-0.02) | |
| Madagascar | -2.97  (-3.23--2.71) | | 288.33  (221.08-367.59) | | -4.32  (-4.55--4.1) | | | 199.93  (15.09-665.83) | | -4.33  (-4.55--4.1) | | 2.3  (0.15-7.71) | |
| Malawi | -2.43  (-2.75--2.11) | | 209.67  (160.01-268.27) | | -2.56  (-2.87--2.25) | | | 215.53  (23.18-673.72) | | -2.57  (-2.88--2.26) | | 2.48  (0.25-7.79) | |
| Malaysia | -1.61  (-2--1.22) | | 221.92  (169.73-283.38) | | -3.9  (-4.35--3.44) | | | 37.4  (4.78-97.86) | | -3.97  (-4.42--3.51) | | 0.42  (0.04-1.12) | |
| Maldives | -1.91  (-3.36--0.45) | | 274.49  (210.66-350.14) | | -5.54  (-6.93--4.14) | | | 43.33  (5.96-125.59) | | -5.66  (-7.04--4.26) | | 0.48  (0.05-1.44) | |
| Mali | -2.03  (-2.41--1.65) | | 496.06  (376.2-634.96) | | -2.41  (-3.06--1.74) | | | 558.73  (55.34-1830.27) | | -2.41  (-3.07--1.75) | | 6.43  (0.62-21.19) | |
| Malta | -3.72  (-4.93--2.5) | | 113.41  (86.13-147.05) | | -2.99  (-4.04--1.93) | | | 1.01  (0.58-1.58) | | 2.48  (1.5-3.46) | | 0  (0-0.01) | |
| Marshall Islands | 4.54  (3.65-5.44) | | 547.05  (413.87-699.33) | | 3.66  (2.63-4.71) | | | 279.86  (29.42-706.12) | | 3.66  (2.63-4.7) | | 3.22  (0.3-8.22) | |
| Mauritania | -1.53  (-1.68--1.39) | | 489.78  (371.7-627.84) | | -3.39  (-3.77--3.01) | | | 197.67  (20.11-690.05) | | -3.41  (-3.79--3.03) | | 2.26  (0.19-7.96) | |
| Mauritius | -0.06  (-0.44-0.32) | | 291.55  (223.68-371.55) | | 0.22  (-0.18-0.62) | | | 2.1  (1.24-3.31) | | 9.86  (8.79-10.94) | | 0  (0-0) | |
| Mexico | -1.03  (-2.12-0.06) | | 419.38  (319.63-535.4) | | -2.38  (-3.93--0.8) | | | 5.44  (3.47-13.12) | | -3.06  (-4.93--1.16) | | 0.03  (0.02-0.11) | |
| Micronesia (Federated States of) | 0.51  (0.21-0.82) | | 513.54  (389.37-657.02) | | -1.42  (-1.74--1.1) | | | 193.64  (25.84-515.61) | | -1.44  (-1.76--1.12) | | 2.22  (0.26-5.96) | |
| Monaco | -0.41  (-0.54--0.29) | | 13.85  (9.52-19.85) | | -1.98  (-2.17--1.79) | | | 1.18  (0.19-3.4) | | -2.09  (-2.29--1.9) | | 0.01  (0-0.04) | |
| Mongolia | -4.47  (-4.73--4.21) | | 140.4  (107.03-181.82) | | -7  (-7.3--6.69) | | | 50.69  (5.33-147.6) | | -7.03  (-7.34--6.73) | | 0.58  (0.05-1.7) | |
| Montenegro | -0.31  (-0.7-0.08) | | 257.75  (197.7-329.03) | | -3.32  (-3.71--2.92) | | | 32.36  (4.93-78.91) | | -3.41  (-3.8--3.01) | | 0.35  (0.04-0.89) | |
| Morocco | -3.22  (-3.69--2.74) | | 138.69  (105.73-179.56) | | -4.59  (-5--4.17) | | | 47.04  (5.88-159.49) | | -4.61  (-5.03--4.2) | | 0.53  (0.06-1.84) | |
| Mozambique | -4.32  (-4.74--3.89) | | 167.65  (128.52-216.93) | | -4.61  (-5.08--4.13) | | | 220.31  (23.66-750.88) | | -4.62  (-5.1--4.14) | | 2.53  (0.25-8.66) | |
| Myanmar | -0.63  (-1.61-0.36) | | 466.82  (354.94-599.1) | | -1.59  (-2.33--0.84) | | | 272.49  (26.81-817.34) | | -1.62  (-2.36--0.87) | | 3.12  (0.28-9.41) | |
| Namibia | -3.18  (-3.64--2.72) | | 204.37  (156.08-262.06) | | -3.23  (-3.83--2.63) | | | 110.83  (10.41-331.64) | | -3.24  (-3.84--2.64) | | 1.27  (0.11-3.82) | |
| Nauru | -6.34  (-7.76--4.89) | | 66.48  (49.37-88.2) | | -6.63  (-8.17--5.06) | | | 32.78  (3.62-97.96) | | -6.64  (-8.18--5.07) | | 0.38  (0.04-1.13) | |
| Nepal | -4.49  (-5.33--3.64) | | 293.44  (225.09-374.05) | | -5.77  (-6.47--5.06) | | | 123.27  (16.06-352.48) | | -5.79  (-6.5--5.08) | | 1.41  (0.17-4.07) | |
| Netherlands | 8.63  (7.49-9.78) | | 128.98  (98.29-166.84) | | 9.12  (8.24-10.01) | | | 1.12  (0.66-1.79) | | 10.77  (8.92-12.64) | | 0  (0-0.01) | |
| New Zealand | -3.19  (-3.35--3.04) | | 149.35  (114.44-193.81) | | -2.32  (-2.52--2.12) | | | 1.25  (0.76-1.9) | | 10.81  (9.1-12.55) | | 0  (0-0.01) | |
| Nicaragua | -12.76  (-14.25--11.25) | | 16.5  (11.44-23.52) | | -15.05  (-16.52--13.57) | | | 4.96  (0.56-16.16) | | -15.09  (-16.56--13.61) | | 0.06  (0.01-0.19) | |
| Niger | -2.41  (-2.77--2.04) | | 528.24  (399.39-675.28) | | -3.49  (-3.95--3.03) | | | 513.53  (43.2-1633.03) | | -3.5  (-3.96--3.04) | | 5.92  (0.46-18.92) | |
| Nigeria | -0.61  (-0.8--0.42) | | 675.92  (508.71-867.69) | | -1.19  (-1.48--0.9) | | | 489.38  (55.17-1585.87) | | -1.2  (-1.49--0.91) | | 5.62  (0.59-18.36) | |
| Niue | 1.21  (-0.19-2.63) | | 246.77  (189.12-315.13) | | -0.78  (-2.02-0.47) | | | 69.44  (9.45-190.16) | | -0.82  (-2.06-0.43) | | 0.79  (0.09-2.19) | |
| North Macedonia | 0.97  (0.12-1.83) | | 261.48  (200.56-333.79) | | -1.29  (-2.21--0.36) | | | 45.25  (6.86-118.07) | | -1.36  (-2.28--0.43) | | 0.5  (0.06-1.34) | |
| Northern Mariana Islands | 2.19  (1.2-3.2) | | 171  (131.28-221.85) | | 2.01  (0.77-3.26) | | | 31.27  (4.21-90.02) | | 2  (0.76-3.27) | | 0.35  (0.03-1.03) | |
| Norway | -2.11  (-2.68--1.53) | | 100.6  (76.16-130.42) | | -1.66  (-2.22--1.1) | | | 0.79  (0.48-1.22) | | 5.99  (5.3-6.69) | | 0  (0-0) | |
| Oman | 0.55 (0-1.12) | | 246.43 (188.81-314.69) | | -2.49 (-3.24--1.73) | | | 39.09 (5.66-111.84) | | -2.56 (-3.32--1.8) | | 0.43 (0.05-1.27) | |
| Pakistan | -1.77  (-1.96--1.59) | | 374.11  (286.05-475.77) | | -2.21  (-2.48--1.94) | | | 183.69  (18.1-518.87) | | -2.23  (-2.5--1.96) | | 2.11  (0.18-6) | |
| Palau | 1.58  (0-3.18) | | 304.94  (233.82-388.76) | | 0.22  (-1.31-1.78) | | | 52.54  (6.84-152.11) | | 0.18  (-1.35-1.74) | | 0.59  (0.06-1.73) | |
| Palestine | -0.31  (-1.87-1.28) | | 63.05  (46.85-83.82) | | -1.79  (-3.49--0.05) | | | 15.93  (1.82-47.44) | | -1.82  (-3.53--0.08) | | 0.18  (0.01-0.54) | |
| Panama | 1.28  (0.8-1.76) | | 349.14  (267.12-443.9) | | -5.06  (-6.08--4.02) | | | 3.13  (1.94-5.03) | | -9.84  (-11.54--8.1) | | 0.01  (0-0.02) | |
| Papua New Guinea | -1.46  (-1.78--1.14) | | 526.09  (398.25-672.55) | | -1.52  (-1.79--1.26) | | | 397.84  (38.3-1159.44) | | -1.53  (-1.79--1.26) | | 4.57  (0.41-13.41) | |
| Paraguay | -3.89  (-4.58--3.2) | | 186.27  (142.52-239.97) | | -5.82  (-6.21--5.43) | | | 45.35  (6.23-124.57) | | -5.87  (-6.26--5.49) | | 0.51  (0.06-1.43) | |
| Peru | -0.61  (-0.96--0.26) | | 383.27  (292.85-488.18) | | -4.3  (-4.66--3.93) | | | 88.99  (10.87-262.17) | | -4.36  (-4.73--3.99) | | 1  (0.09-3.01) | |
| Philippines | -0.72  (-1.13--0.3) | | 374.34  (286.19-476.18) | | -1.5  (-1.91--1.08) | | | 141.46  (15.77-368.54) | | -1.51  (-1.93--1.09) | | 1.61  (0.15-4.24) | |
| Poland | -3.04  (-3.87--2.19) | | 115.31  (87.73-149.24) | | -3.42  (-4.23--2.6) | | | 0.97  (0.63-1.47) | | -4.51  (-5.38--3.62) | | 0  (0-0) | |
| Portugal | -2.38  (-2.95--1.82) | | 89.32  (67.3-117.07) | | -0.73  (-1.44--0.02) | | | 0.86  (0.49-1.36) | | 10.91  (7.78-14.14) | | 0  (0-0.01) | |
| Puerto Rico | -2  (-2.38--1.61) | | 259.38  (198.75-331.23) | | -1.56  (-1.93--1.2) | | | 1.95  (1.21-3.03) | | 7.45  (6.29-8.63) | | 0  (0-0) | |
| Qatar | -2.21  (-3.52--0.89) | | 167.09  (127.77-215.75) | | -6.08  (-7.36--4.78) | | | 21.97  (3.14-60.88) | | -6.2  (-7.47--4.91) | | 0.24  (0.02-0.7) | |
| Republic of Korea | -5.6  (-6.59--4.59) | | 95.48  (72.03-123.92) | | -8.82  (-9.58--8.04) | | | 8.73  (1.55-24.06) | | -8.98  (-9.74--8.21) | | 0.09  (0.01-0.27) | |
| Republic of Moldova | -1.49  (-2.69--0.28) | | 219.92  (167.9-281.34) | | -1.28  (-2.5--0.04) | | | 1.6  (0.97-2.5) | | 3.03  (1.62-4.47) | | 0  (0-0) | |
| Romania | 3.88  (2.36-5.42) | | 288.85  (221.47-368.32) | | 2.25  (0.25-4.29) | | | 2.12  (1.28-3.28) | | -4.36  (-8.04--0.53) | | 0  (0-0) | |
| Russian Federation | -14.81  (-16.12--13.49) | | 14.88  (10.23-21.31) | | -10.54  (-11.6--9.46) | | | 0.34  (0.18-0.47) | | -5.1  (-5.81--4.39) | | 0  (0-0) | |
| Rwanda | -5.42  (-6.23--4.6) | | 101.74  (76.95-131.89) | | -5.71  (-6.66--4.75) | | | 98.23  (9.81-319.48) | | -5.72  (-6.68--4.76) | | 1.13  (0.11-3.69) | |
| Saint Kitts and Nevis | 6.49  (5.45-7.53) | | 141.19  (108.01-183.11) | | 0.08  (-0.71-0.87) | | | 1.51  (0.95-2.29) | | -4.25  (-5.68--2.79) | | 0.01  (0-0.01) | |
| Saint Lucia | 1.95  (0.69-3.22) | | 237.33  (181.53-303.06) | | -0.56  (-1.29-0.16) | | | 2.5  (1.54-3.98) | | -3.24  (-4.58--1.88) | | 0.01  (0.01-0.02) | |
| Saint Vincent and the Grenadines | 5.28  (3.93-6.65) | | 161.22  (123.77-209.22) | | -0.71  (-1.43-0.02) | | | 1.59  (0.97-2.44) | | -5.38  (-6.64--4.11) | | 0.01  (0-0.01) | |
| Samoa | -1.64  (-2.02--1.26) | | 440.3  (332.26-564.05) | | -4.61  (-4.89--4.34) | | | 76.66  (11.48-219.12) | | -4.64  (-4.92--4.37) | | 0.87  (0.1-2.54) | |
| San Marino | 7.58  (6.92-8.25) | | 239.18  (183.33-305.42) | | 5.25  (4.68-5.82) | | | 19.28  (3.3-50.99) | | 5.09  (4.53-5.66) | | 0.2  (0.02-0.57) | |
| Sao Tome and Principe | -4.2  (-4.71--3.7) | | 164.11  (125.93-212.83) | | -5.67  (-6.51--4.84) | | | 119.43  (11.22-347.53) | | -5.69  (-6.52--4.85) | | 1.37  (0.12-4.01) | |
| Saudi Arabia | -4.36  (-4.66--4.07) | | 86.13  (64.71-113.01) | | -7.12  (-7.51--6.73) | | | 17.35  (2.28-50.04) | | -7.18  (-7.58--6.79) | | 0.19  (0.02-0.57) | |
| Senegal | -4.14  (-4.51--3.77) | | 231.96  (177.42-296.18) | | -4.44  (-4.84--4.05) | | | 205.74  (18.77-635.69) | | -4.45  (-4.84--4.06) | | 2.37  (0.2-7.34) | |
| Serbia | -0.44  (-0.93-0.06) | | 294.44  (225.94-375.18) | | -4.69  (-5.25--4.13) | | | 34.32  (5.45-87.28) | | -4.83  (-5.39--4.27) | | 0.37  (0.04-0.98) | |
| Seychelles | -1.37  (-2.19--0.54) | | 246.47  (188.78-314.74) | | -2.16  (-2.81--1.51) | | | 55.17  (8.46-153.14) | | -2.2  (-2.85--1.55) | | 0.62  (0.08-1.76) | |
| Sierra Leone | -2.15  (-2.61--1.69) | | 317.75  (243.94-404.64) | | -2.51  (-2.84--2.19) | | | 416.23  (33.3-1340.95) | | -2.52  (-2.84--2.2) | | 4.78  (0.36-15.45) | |
| Singapore | -1.55  (-2.09--1.01) | | 170.15  (130.39-220.26) | | -1.05  (-1.62--0.48) | | | 1.29  (0.78-2.01) | | 12.1  (10.57-13.66) | | 0  (0-0) | |
| Slovakia | 8.96  (8.12-9.82) | | 139.88  (106.95-181.28) | | 6.25  (5.36-7.14) | | | 16.88  (2.34-48.27) | | 6.14  (5.25-7.03) | | 0.18  (0.02-0.55) | |
| Slovenia | 7.87  (5.98-9.8) | | 174.85  (133.93-226.09) | | 7.82  (6-9.67) | | | 1.25  (0.73-1.99) | | 6.29  (5.16-7.43) | | 0  (0-0) | |
| Solomon Islands | -1.73  (-1.85--1.6) | | 292.12  (223.96-372.47) | | -2.23  (-2.43--2.04) | | | 137.24  (13.99-376.24) | | -2.24  (-2.44--2.04) | | 1.58  (0.14-4.38) | |
| Somalia | 0.03  (-0.28-0.34) | | 799.14  (598.34-1018.99) | | 0.23  (-0.17-0.63) | | | 953.97  (114.57-2869.82) | | 0.23  (-0.17-0.64) | | 10.99  (1.26-33.36) | |
| South Africa | 1.14  (0.94-1.33) | | 519.8  (394.04-664.77) | | -0.97  (-1.24--0.7) | | | 177.67  (17.84-468.67) | | -1  (-1.28--0.73) | | 2.02  (0.17-5.39) | |
| South Sudan | -0.58  (-0.93--0.24) | | 557.79  (420.92-714.6) | | -0.9  (-1.19--0.6) | | | 639.08  (61.09-2132.29) | | -0.89  (-1.19--0.6) | | 7.35  (0.66-24.62) | |
| Spain | -2.8  (-3.95--1.64) | | 160.65  (123.34-208.47) | | 0.77  (-0.5-2.06) | | | 1.88  (1.01-3.06) | | 21.53  (18.79-24.34) | | 0.01  (0-0.02) | |
| Sri Lanka | 4.32  (1.99-6.71) | | 121.36  (92.32-157.15) | | 2.1  (-0.1-4.35) | | | 20.46  (2.64-59.04) | | 2.02  (-0.17-4.27) | | 0.23  (0.02-0.68) | |
| Sudan | -2.3  (-2.6--1.99) | | 411.67  (314.17-525.61) | | -3.61  (-4.05--3.17) | | | 163.44  (17.02-507.98) | | -3.63  (-4.06--3.19) | | 1.87  (0.18-5.83) | |
| Suriname | -5.3  (-5.64--4.97) | | 91.92  (69.28-120.36) | | -5.9  (-6.33--5.47) | | | 34.44  (3.92-111.19) | | -5.91  (-6.34--5.48) | | 0.39  (0.04-1.28) | |
| Sweden | 0.4  (-0.13-0.93) | | 75.22  (56.36-99.05) | | 1.21  (0.85-1.58) | | | 0.65  (0.39-1.01) | | 7.9  (6.12-9.71) | | 0  (0-0) | |
| Switzerland | -2.73  (-3.36--2.1) | | 78.05  (58.46-102.62) | | -1.88  (-2.41--1.34) | | | 0.64  (0.38-1.01) | | 10.78  (9.57-12) | | 0  (0-0) | |
| Syrian Arab Republic | 3.07  (1.98-4.17) | | 570.74  (430.7-730.65) | | 1.06  (-0.19-2.32) | | | 131.54  (15.31-359.57) | | 1.03  (-0.22-2.3) | | 1.48  (0.13-4.13) | |
| Taiwan (Province of China) | -3.54  (-3.85--3.23) | | 93.52  (70.5-122.42) | | -2.8  (-3.13--2.47) | | | 0.75  (0.44-1.19) | | 12.06  (10.83-13.3) | | 0  (0-0) | |
| Tajikistan | -3.74  (-4.15--3.33) | | 189.9  (145.42-244.45) | | -3.23  (-3.63--2.84) | | | 112.93  (13.95-329.91) | | -3.24  (-3.63--2.84) | | 1.29  (0.15-3.8) | |
| Thailand | -0.27  (-0.96-0.42) | | 239.97  (183.56-306.43) | | -2.83  (-3.37--2.28) | | | 44.13  (5.34-122.1) | | -2.9  (-3.44--2.36) | | 0.49  (0.04-1.4) | |
| Timor-Leste | -2.84  (-3.25--2.44) | | 514.86  (390.04-658.2) | | -4.14  (-4.54--3.74) | | | 245.15  (25.49-696.27) | | -4.16  (-4.56--3.75) | | 2.81  (0.26-8.03) | |
| Togo | -1.77  (-2.47--1.06) | | 298.37  (228.88-380.35) | | -3.33  (-4.04--2.62) | | | 169.7  (18.95-568.27) | | -3.34  (-4.05--2.63) | | 1.95  (0.2-6.58) | |
| Tokelau | -1.07  (-1.47--0.67) | | 288.19  (220.93-367.45) | | -3.8  (-4.19--3.41) | | | 70.52  (8.98-196.67) | | -3.85  (-4.24--3.45) | | 0.8  (0.09-2.26) | |
| Tonga | 8.26  (5.53-11.05) | | 496.12  (376.56-636.11) | | 6.84  (4.44-9.3) | | | 164.84  (22.32-421.25) | | 6.83  (4.43-9.28) | | 1.87  (0.21-4.85) | |
| Trinidad and Tobago | -1.8  (-2.12--1.47) | | 402.08  (306.85-513.36) | | -1.72  (-2.04--1.4) | | | 2.82  (1.64-4.49) | | 0.84  (0.15-1.54) | | 0  (0-0) | |
| Tunisia | -3.33  (-4.55--2.1) | | 128.64  (97.93-166.3) | | -6.95  (-8.09--5.8) | | | 21.41  (2.57-63.76) | | -7.03  (-8.16--5.88) | | 0.24  (0.02-0.73) | |
| Turkey | -5.81  (-6.97--4.63) | | 100.73  (75.93-130.82) | | -9.45  (-10.53--8.34) | | | 20.93  (2.6-62.52) | | -9.52  (-10.61--8.41) | | 0.23  (0.02-0.71) | |
| Turkmenistan | -8.39  (-9.13--7.64) | | 31.19  (22.39-42.89) | | -0.84  (-1.91-0.23) | | | 1.84  (0.54-3.56) | | 8.12  (5.4-10.91) | | 0.02  (0.01-0.04) | |
| Tuvalu | -1.19  (-1.34--1.04) | | 507.21  (383.82-648.41) | | -3.67  (-3.85--3.48) | | | 175.6  (21.92-499.55) | | -3.69  (-3.88--3.51) | | 2  (0.21-5.79) | |
| Uganda | -1.97  (-2.2--1.74) | | 364.93  (279.2-463.97) | | -1.57  (-1.92--1.22) | | | 273.82  (29.58-831.16) | | -1.57  (-1.92--1.22) | | 3.15  (0.31-9.64) | |
| Ukraine | 6.05  (1.96-10.3) | | 624.72  (471.49-800.44) | | 4.39  (1.57-7.29) | | | 4.66  (2.79-7.24) | | 0.44  (-0.79-1.69) | | 0  (0-0.01) | |
| United Arab Emirates | -2.55  (-2.94--2.15) | | 121.74  (92.48-157.85) | | -4.65  (-5.01--4.28) | | | 31.01  (3.48-85.5) | | -4.68  (-5.05--4.32) | | 0.35  (0.03-0.99) | |
| United Kingdom | -2.86  (-3.44--2.28) | | 119.9  (91.29-154.96) | | -0.99  (-1.55--0.43) | | | 1.38  (0.87-1.94) | | 5.5  (4.18-6.84) | | 0.01  (0-0.01) | |
| United Republic of Tanzania | -2.3  (-2.57--2.03) | | 225.4  (172.22-288.08) | | -1.21  (-1.53--0.89) | | | 279.14  (29.94-817.18) | | -1.22  (-1.54--0.9) | | 3.21  (0.33-9.44) | |
| United States of America | 0.55  (-0.17-1.28) | | 171.88  (131.71-222.33) | | 1.17  (0.62-1.71) | | | 1.61  (1.08-2.35) | | 3.11  (2.05-4.18) | | 0  (0-0.01) | |
| United States Virgin Islands | -0.15  (-0.8-0.5) | | 551.39  (417.14-704.89) | | -3  (-3.52--2.48) | | | 85.3  (13.51-214.97) | | -3.07  (-3.58--2.56) | | 0.95  (0.11-2.46) | |
| Uruguay | -0.64  (-0.95--0.32) | | 208.53  (159.12-266.95) | | 3.31  (2.16-4.48) | | | 3.02  (1.43-5.6) | | 14.05  (9.54-18.75) | | 0.02  (0-0.05) | |
| Uzbekistan | -0.7  (-1.44-0.04) | | 157.77  (120.97-204.75) | | -2.25  (-2.72--1.78) | | | 1.38  (0.87-2.14) | | -5.2  (-5.94--4.47) | | 0.01  (0-0.01) | |
| Vanuatu | -0.02  (-0.3-0.27) | | 618.57  (466.16-793.74) | | -0.06  (-0.29-0.17) | | | 307.98  (33.65-841.97) | | -0.06  (-0.29-0.17) | | 3.54  (0.34-9.79) | |
| Venezuela (Bolivarian Republic of) | -0.38  (-0.71--0.06) | | 647.42  (488.55-829.33) | | -0.82  (-1.23--0.4) | | | 10.25  (5.02-17.43) | | -1.06  (-1.7--0.42) | | 0.07  (0.01-0.15) | |
| Viet Nam | -0.56  (-0.81--0.32) | | 356.04  (272.49-452.97) | | -2.34  (-2.55--2.14) | | | 82.98  (11.43-219.32) | | -2.39  (-2.6--2.18) | | 0.93  (0.11-2.51) | |
| Yemen | -1.18  (-1.5--0.85) | | 555.76  (420.3-710.47) | | -2.15  (-2.5--1.79) | | | 196.66  (21.79-601.4) | | -2.16  (-2.51--1.81) | | 2.24  (0.21-6.95) | |
| Zambia | -2.38  (-2.74--2.02) | | 176.65  (135.16-228.2) | | -3.86  (-4.38--3.34) | | | 148.72  (16.86-500.45) | | -3.87  (-4.39--3.35) | | 1.71  (0.18-5.77) | |
| Zimbabwe | 0.85  (-0.19-1.91) | | 323.41  (248.25-411.83) | | 3.93  (3-4.88) | | | 239.95  (22.09-719.69) | | 3.98  (3.04-4.93) | | 2.76  (0.22-8.32) | |

Abbreviations: ASIR, age-standardized incidence rate; ASDR, age-standardized death rate; DALYs, disability-adjusted life years; ASYR age-standardized DALYs rate; EAPC, estimated annual percentage change
