# Supplementary material for: Global burden of pertussis in 204 countries and territories, from 1990 to 2019: results from the Global Burden of Disease Study 2019
Source: BMC Public Health. 2024 May 30;24:1453. doi: 10.1186/s12889-024-18968-y (PMC11141049; doi:10.1186/s12889-024-18968-y)
Supplement: Supplementary file 5 — Supplementary Material 5. [file 12889_2024_18968_MOESM5_ESM.docx]

Table S4 Normality test of pertussis related indicators

| Variables | *D* value | *P* value |
| --- | --- | --- |
| SDI | 0.074 | 0.009 |
| ASIR | 0.104 | < 0.001 |
| ASYR | 0.257 | < 0.001 |
| ASDR | 0.259 | < 0.001 |
| EAPC of ASIR | 0.111 | < 0.001 |
| EAPC of ASYR | 0.098 | < 0.001 |
| EAPC of ASDR | 0.166 | < 0.001 |
